# Faster and Durable: A Cell‐to‐System Validation of a Low‐Degradation Fast‐Charge Protocol for Li‐Ion Batteries

**DOI:** 10.1002/advs.75306

**Published:** 2026-04-17

**Authors:** Marco Lagnoni, Francesco Giuseppe Quilici, Davide Cademartori, Claudio Scarpelli, Uways Nurulain Mithoowani, Federica Barontini, Alessandro Ruvio, Monica Puccini, Giovanni Lutzemberger, Maria Paola Carpanese, Antonio Bertei

**Affiliations:** ^1^ Department of Civil and Industrial Engineering University of Pisa Pisa Italy; ^2^ Department of Energy Systems Territory and Constructions Engineering University of Pisa Pisa Italy; ^3^ Department of Civil Chemical and Environmental Engineering University of Genoa Genova Italy; ^4^ Department of Electrical and Energy Engineering Sapienza University of Rome Rome Italy

## Abstract

Fast charging of Li‐ion batteries remains a critical barrier for electric vehicle adoption due to parasitic reactions at the negative electrode, which irreversibly reduce lithium inventory, closely linked to graphite phase separation dynamics. This study presents a physics‐based fast‐charge protocol derived from a pseudo‐two‐dimensional phase‐field electrochemical model that was rigorously calibrated and validated through teardown characterisation, galvanostatic cycling, and impedance spectroscopy on a commercial cylindrical battery. The model‐informed protocol achieves charging from 20% to 80% state‐of‐charge within 15 min, outperforming both the manufacturer specification (18 min) and a commercial electric vehicle profile (25 min), while limiting capacity loss below 5% over 500 cycles. System‐level simulations of a charging station demonstrate compatibility with current electric infrastructure and show significant improvements in throughput and users waiting time under realistic usage scenarios. The work demonstrates that exploiting internal cell physics through model‐informed control can unlock high‐rate charging without hardware modification, offering a viable path toward low‐degradation fast charging.

## Graphical abstract

A physics‐based framework resolving graphite phase‐separation dynamics establishes a predictive, degradation‐aware fast‐charging methodology for commercial Li‐ion batteries. The resulting model‐informed protocol achieves 20%–80% state‐of‐charge in 14 min while matching the long‐term degradation of a commercial 25‐minute EV strategy. Cell‐to‐system validation demonstrates performance gains enabled by physics‐informed control of internal electrochemical states.

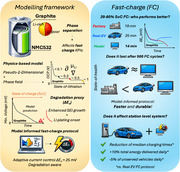

## 1 Introduction

Lithium‐ion batteries are key to global efforts aimed at decarbonising the transport sector through electrification. Although lithium‐ion battery technology is mature and commercially well‐established, the average effective charging duration still remains approximately one hour, significantly longer than the few minutes required to refuel traditional combustion engine vehicles [1]. Consequently, achieving fast‐charge, defined as reaching 80% state‐of‐charge (SoC) in 15 min or less [2], is a critical milestone to enhance the widespread adoption of electric vehicles (EVs), as explicitly targeted by organisations such as the U.S. Advanced Battery Consortium (USABC) [3] and supported by recent studies on charging strategies [4].

While fast‐charge capability is highly desirable from both commercial and consumer perspectives, it presents considerable challenges related to battery degradation [5, 6]. Typical degradation mechanisms widely reported in lithium‐ion cells include active‐material structural deterioration, electrolyte decomposition and dry out [7], electrode particle cracking [8], and lithium inventory loss [9]. Among these, phenomena occurring at graphite‐based negative electrodes, which are prevalent in commercial lithium‐ion batteries, are particularly critical during fast‐charge [10]. Specifically, graphite electrodes are highly susceptible to solid–electrolyte interphase (SEI) growth [11, 12] and lithium plating [13]. SEI growth is an inevitable degradation process that irreversibly consumes lithium ions via reactions between lithium and electrolyte solvents, typically causing little capacity fade per cycle [14]. Lithium plating becomes especially critical under severe fast‐charge conditions, occurring when the local potential at the graphite surface falls below 0 V versus Li/Li^+^, causing lithium ions to deposit as metallic lithium rather than intercalating into graphite particles [15]. This metallic lithium is highly reactive and can rapidly induce severe irreversible capacity loss via formation of additional SEI or in case electrical contact is lost [16]. Furthermore, graphite phase‐separation behavior upon intercalation [17], known as staging, strongly influences these degradation processes [18]. During staging, graphite separates into distinct stable phases with different lithium stoichiometry (i.e., LiC_6_, LiC_12_, LiC_18_), directly influencing local electrode potentials and significantly affecting the propensity for both SEI growth and lithium plating [19]. Therefore, detailed consideration of graphite staging behavior is essential to accurately prevent and mitigate battery degradation under fast‐charge conditions.

Commercially available batteries typically restrict recommended fast‐charge rates by the manufacturer to a maximum of 2C (equivalent to a 30‐minute full charge) to avoid significant degradation and preserve battery longevity [20]. Electric vehicle manufacturers are generally more conservative: although they may allow higher current peaks, they keep the average C‐rate below 2C to further reduce long‐term ageing [21]. Despite these precautions, achieving the target of approximately 80% SoC in 15 min necessitates carefully designed protocols capable of dynamically balancing fast‐charge rates against battery longevity. Simply increasing current densities to reduce charging times leads to significantly enhanced degradation, whereas overly conservative protocols fail to meet consumer expectations.

Numerous fast‐charge strategies have been investigated in recent years to address the critical challenge of lithium plating and battery degradation [21]. These approaches include constant‐current constant‐voltage, multi‐stage constant‐current [22, 23], pulsed‐current charging [24, 25], and dynamically modulated charging profiles [26, 27]. Each of these protocols aims to adjust the applied current to mitigate the onset of lithium plating, typically relying on reducing currents at higher states of charge [28]. For instance, multi‐stage constant‐current and pulse charging protocols dynamically adjust the current to maintain the negative electrode potential above critical thresholds, thus minimizing lithium plating propensity [29]. While some studies have explored thermal modulation to enhance kinetics and accelerate charging [30, 31], practical limitations such as safety concerns, infrastructure demands, and accelerated ageing have so far hindered widespread implementation [32]. As such, current research continues to focus on current‐based protocols that are more readily deployable and controllable under typical EV operating conditions [33].

To effectively unlock fast‐charging capabilities, physics‐based multi‐scale battery models have emerged as essential tools to engineer cell design and optimize charging protocols through a quantitative understanding of internal states and degradation pathways under high‐rate operation [18, 34, 35, 36, 37, 38]. Several types of models have been developed with different levels of complexity, ranging from single‐particle models to 3D microstructure‐resolved approaches [39, 40]. Single‐particle models are computationally efficient but cannot capture the through‐thickness transport and local potential variations that govern lithium plating during fast charge. Fully resolved 3D models are accurate but remain prohibitively expensive for systematic protocol design [41]. Pseudo‐two‐dimensional (P2D) models strike a favorable balance: they explicitly resolve both the through‐thickness direction and the radial particle coordinate, enabling the prediction of spatially non‐uniform lithium concentrations as well as electronic and ionic potentials across the cell, which directly influence plating propensity. When rigorously parameterized and validated, P2D models have been successfully employed to design fast‐charge strategies and mitigate lithium plating [21, 26, 28, 29]. Recent comparative studies further demonstrate that P2D and microstructure‐resolved 3D simulations yield near‐equivalent cell‐level predictions over practical C‐rates (up to 5C) for realistic electrode architectures, with significant discrepancies emerging only under extreme porosity, very high tortuosity or unrealistically high rates (>7C) [42, 43]. These findings, consistent with prior protocol‐design efforts, support the adoption of the P2D framework as the appropriate level of fidelity for generalizable and computationally efficient fast‐charging protocol development, while reserving targeted 3D analyses for local inhomogeneity or ageing studies.

To overcome the limitations of standard P2D models in capturing the complex intercalation physics of graphite electrodes, phase‐field formulations have recently been integrated into multi‐scale frameworks [15, 18, 19, 44]. Unlike conventional approaches based on Fick diffusion [45], which assume smooth concentration profiles, phase‐field models based on Cahn‐Hilliard non‐equilibrium thermodynamics explicitly account for the intrinsic phase‐separation behavior of graphite, in which intercalated lithium phase‐separates into Li‐rich and Li‐poor domains [46, 47, 48]. When properly calibrated, a phase‐field model enables the accurate prediction of uphill diffusion, spinodal decomposition, intercalation wave versus shrinking core structures [49], which represent key features of staging dynamics. As a result, phase‐field modelling offers a reliable mechanistic insight into lithium intercalation and allows accurate tracking of otherwise inaccessible internal variables, such as local electrode potentials and lithium distribution, thus providing a robust foundation for the design of advanced model‐informed charging protocols [17, 18].

Hence, the effective implementation of modelling approaches to engineer fast‐charging requires three fundamental elements [50]: (i) a comprehensive physics‐based phase‐field model, accounting for all key electrochemical and thermodynamic processes; (ii) a rigorous parameter calibration, derived from cell teardown coupled with multiple electrochemical characterizations; and (iii) a thorough validation, achieved through extensive experimental cycling tests to translate model predictions into practical charging strategies.

This study presents a physics‐based fast‐charge strategy informed by a P2D non‐equilibrium phase‐field framework to account for graphite phase‐separation dynamics during lithium intercalation. The model is used to design and optimize a fast‐charge protocol capable of recharging a commercial 18650 lithium‐ion cell to 80% state‐of‐charge (SoC) in less than 15 min, through dynamic modulation of the applied current based on continuous monitoring of the local potential difference at the negative electrode. Notably, the latter is used solely within the model during protocol design, so that the implementation of the protocol itself requires only power vs state‐of‐charge master curves and no additional sensing. Model parameterization is supported by an extensive teardown and electrochemical characterization campaign, enabling a statistically representative estimation of parameter distributions across all key physical and transport properties. Additional parameter refinement is provided through frequency‐domain analysis via electrochemical impedance spectroscopy (EIS). The optimized protocol is benchmarked against both the maximum charging rate indicated by the manufacturer and protocols effectively applied in EVs for this battery chemistry. All three protocols are then experimentally validated through extended cycling over 500 full charge–discharge cycles, with direct measurement of capacity retention to assess battery ageing. Finally, the broader impact of protocol implementation at the charging infrastructure level is evaluated via Monte Carlo simulations by integrating field usage statistics from public fast‐charging stations. The results identify potential system‐level benefits and operational considerations, providing a practical pathway toward integrating model‐informed fast‐charge protocols into real‐world commercial EV platforms.

## 2 Results

### 2.1 Teardown Characterization, Electrical Tests, and Model Calibration

Designing fast‐charging protocols for lithium‐ion batteries requires a reliable thermo‐electrochemical model, accurately parametrized to capture cell geometry, microstructure, and electrochemical behavior. In this study, the cell under investigation is the 2 Ah Samsung INR18650‐20R, comprising graphite at the negative electrode and nickel–manganese–cobalt oxide (NMC532) at the positive electrode, being it representative of commercial EV battery technology. Key model parameters can be broadly categorized into: (i) geometric, compositional, and microstructural parameters, and (ii) physicochemical parameters describing species transport and kinetics. The former are determined through systematic teardown characterization, ensuring statistical relevance by analyzing multiple pristine cells. The latter category includes material‐specific parameters which are in part sourced from consolidated literature data (e.g., electrolyte properties) and, for the missing ones, specifically fitted from dedicated electrochemical tests.

Teardown analyses dedicated to geometric and compositional parameters are performed under an inert atmosphere, as described in the Methods section and in Figures S7–S11. Key results, directly relevant for materials identification, include active material loadings (12.3 ± 0.1 mg cm^−2^ positive, 7.3 ± 0.5 mg cm^−2^ negative), electrode thicknesses (44 µm positive, 51 µm negative), electrode porosities (∼28%), and median particle diameters (10.4 ± 0.2 µm positive, 16.8 ± 0.9 µm negative). Binder and conductive carbon mass fractions (positive: 2.65% ± 0.17% binder, 1.96% ± 0.05% conductive carbon; negative: 2.58% ± 0.32% binder) are revealed via a combination of thermogravimetric (TGA) and inductively coupled plasma optical emission spectroscopy (ICP‐OES) analyses on both electrodes. Additionally, electrode state‐of‐lithiation (${\tilde{c}}_{s}$) at fully charged and discharged conditions are quantified via a combination of ICP‐OES and electrochemical characterisations (see Figure S10), yielding values of (0.294 ± 0.010) − (0.907 ± 0.007) and (0.022 ± 0.001) − (0.799 ± 0.028) for positive and negative electrodes, respectively. Initial irreversible lithium loss associated with SEI formation, determined via ICP‐OES, corresponds to approximately 9% of nominal cell capacity [51]. These key microstructural parameters, summarized in Table S4, form a robust statistical basis for subsequent model calibration.

Physicochemical parameters, which include transport properties and reaction kinetics, are partially adopted from consolidated literature references (e.g., electrolyte properties [52, 53]), while others (e.g., solid‐phase diffusion coefficients, kinetic constants) are initialized from the literature or previous group data and further refined by fitting to targeted galvanostatic cycling and EIS tests. We refer the reader to Table S4 for the complete set of parameters. To rigorously define the 0%–100% SoC window, a constant‐voltage (CV) step is added at the end of both charge and discharge at different C‐rates (defined based on 2 Ah nominal capacity) as shown in Figure S1, enforcing via coulomb counting that the same accessible capacity (*Q_M_*) is cycled in each test during charge and discharge (see Methods for details). This procedure ensures that electrodes consistently reach the same initial and final states of lithiation in all the tests. Galvanostatic tests performed at multiple currents (0.2–4 A) then provide a broad coverage of the battery operating range, supporting robust parameter identification and reducing the risk of overfitting or parameter cross‐correlation. To further reduce potential ambiguity in parameter fitting [54], EIS is conducted across a broad frequency range (1 MHz–0.05 Hz) at multiple SoCs (20%, 35%, 50%, 65%, 80%). EIS enables the separation of overpotentials related to charge‐transfer kinetics, electrolyte transport, and solid‐phase diffusion processes according to their characteristic frequency, enhancing the robustness and uniqueness of the parameter calibration. Overall, this approach seeks to minimize the ambiguous attribution of fitted parameters to distinct electrochemical phenomena.

**FIGURE 1:**
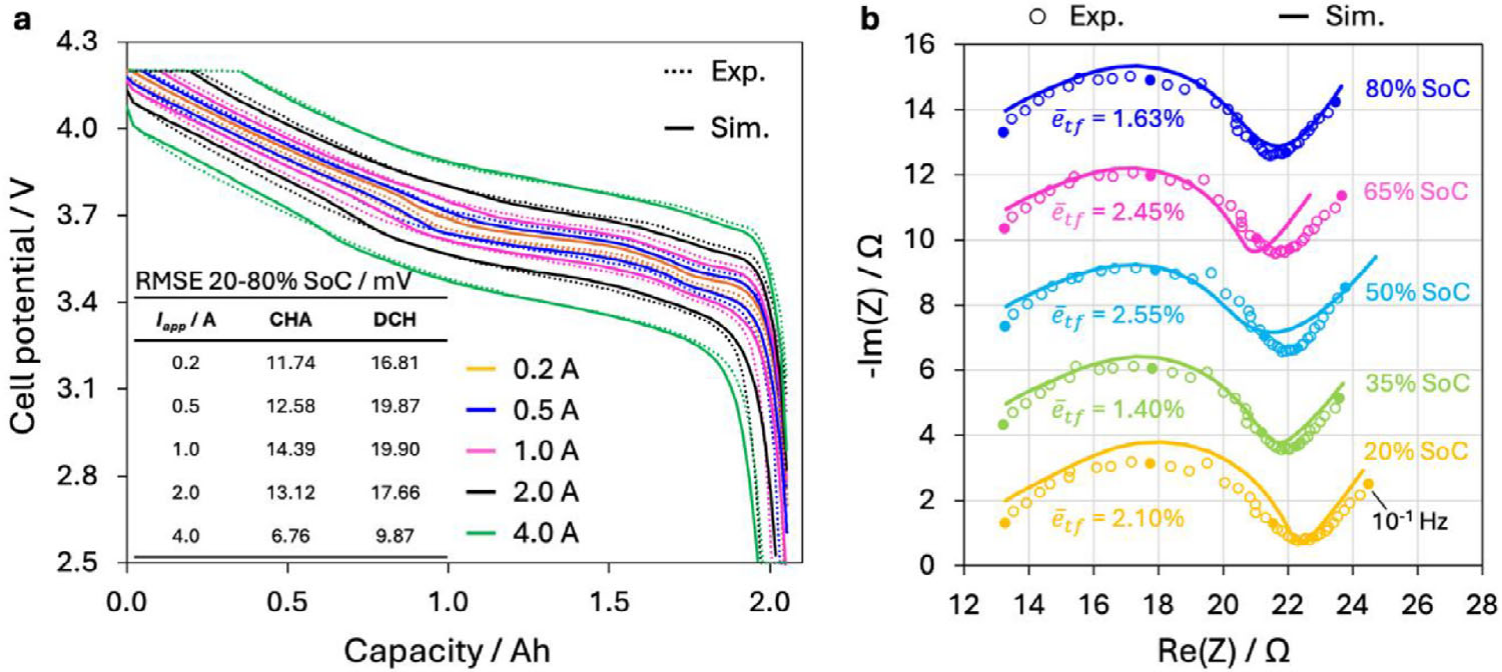
Model calibration using constant current and impedance tests. (a) Comparison of model‐predicted (solid lines) and experimental (dotted lines) charge and discharge curves at different currents. The accompanying table reports the root mean square error for each applied current (0.2–4 A) during charge (CHA) and discharge (DCH). (b) Comparison of model‐predicted (solid lines) and experimental (circles) impedance spectra within 10^−1^–10^3^ Hz at different states of charge (20%–80% SoC with 15% increments). Except for 20% SoC, Nyquist plots are shifted by an imaginary constant for visual clarity.

Figure 1 summarizes the results of the model calibration, reporting a satisfactory agreement between simulations and experimental measurements across multiple galvanostatic cycling conditions and impedance spectra.

Figure 1a reports the comparison between model simulations (solid lines) and experimental voltage profiles (dotted lines) for galvanostatic charge and discharge cycles performed at five current levels: 0.2, 0.5, 1, 2, and 4 A, corresponding to a 0.1–2C range. Across this operational window, the model consistently reproduces the experimental response, accurately tracking the voltage evolution throughout each cycle. The inset reports the root mean square error (RMSE) for both charge (CHA) and discharge (DCH) steps, calculated within the 20%–80% state‐of‐charge (SoC) window relevant for fast‐charging, which excludes the steeper open‐circuit potential gradients that typically arise near full charge or deep discharge [55]. RMSE values remain below 20 mV across all tested conditions, with charging errors ranging from 6.8 to 14.4 mV and discharging errors from 9.9 to 19.9 mV. These results confirm that the model reliably captures the interplay between reaction kinetics and transport phenomena, even under the high current densities representative of practical fast‐charging scenarios.

Complementary validation by EIS (Figure 1b) confirms model fidelity across SoCs and frequency range. Model predictions reproduce the shape and features of the experimental spectra across the 1 kHz–0.1 Hz range, including the high‐frequency semicircle (interfacial charge‐transfer), mid‐frequency feature (electrolyte transport), and low‐frequency tail (solid‐phase diffusion). Open circles denote experimental data, while filled markers highlight each decade in frequency. Relative impedance errors (${\bar{e}}_{tf}$) averaged approximately 2%, confirming model accuracy. Slight discrepancies, observed at very high frequencies (inductive effects from the jelly‐roll configuration [56]) and low frequencies (single‐radius particle assumption for solid‐phase diffusion [57]), do not significantly impact model predictions in practically relevant regimes. Additional validation of thermal responses and frequency‐resolved impedance behaviors further supports model fidelity (Figure S2).

The calibrated model thus integrates statistically relevant geometric and compositional parameters from teardown analysis with robust electrochemical and transport parameters obtained through multiple galvanostatic and impedance tests, yielding a high‐fidelity framework. Notably, fitted parameters (tortuosity and kinetic constants) inherently carry uncertainties, explicitly quantified using a jackknife resampling procedure [58, 59]. To comprehensively assess how these uncertainties affect model predictions under realistic fast‐charging conditions, the next section focuses on model validation against benchmark protocols, including manufacturer‐recommended and commercial EV strategies.

### 2.2 Model Validation Against Manufacturer and Commercial Fast‐Charge Protocols

The cell selected for this study is commercially mature and high‐performing, with demonstrated high‐rate capability and strong capacity retention even under continuous 2C charging (as shown in Figure 1). As such, it represents a high‐standard and challenging test case for fast‐charge protocol development: any improvement must be achieved starting from an already efficient and robust baseline.

Benchmark fast‐charging protocols provide a critical reference for evaluating new model‐informed charging strategies. Beyond serving as experimental benchmarks, commercial protocols offer valuable test cases for assessing the predictive accuracy and robustness of the model parameterization described in the previous section. Macro‐scale indicators, such as cell potential and temperature, which are easily measurable under real operating conditions, provide direct validation of model fidelity. Conversely, micro‐scale indicators, such as local electrode potentials, are relevant to assess degradation propensity but are experimentally inaccessible; thus, their investigation relies entirely on model prediction. To thoroughly validate the model under representative high‐rate conditions, simulations are conducted within 20%–80% state‐of‐charge window targeted by EV fast‐charging strategies.

**FIGURE 2:**
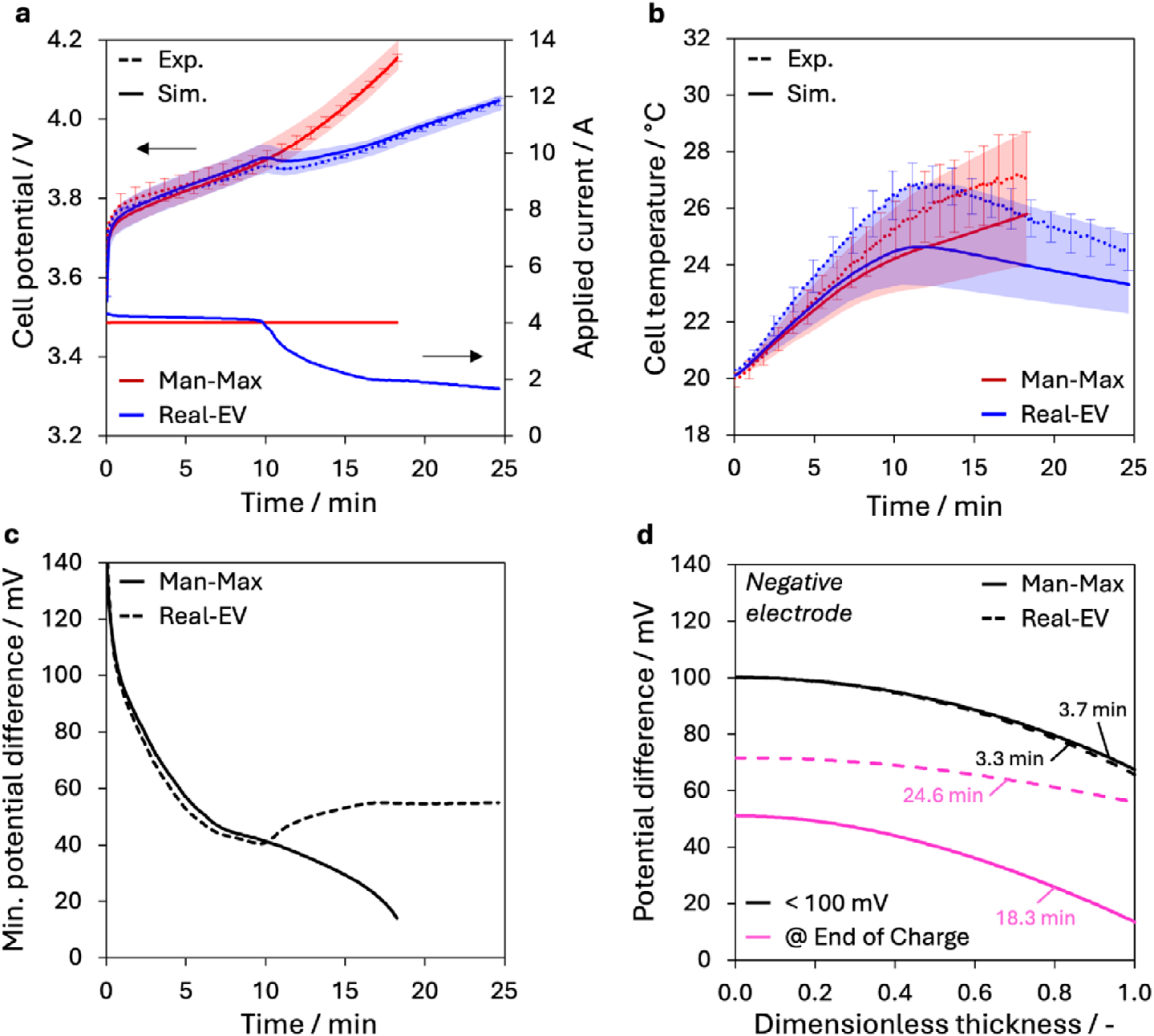
Model validation and comparison of manufacturer and real EV fast‐charge protocols based on macro‐ and micro‐scale electrochemical indicators. (a) Comparison of simulated and experimental battery potential and (b) temperature evolution for two fast‐charge protocols applied to the investigated battery under real‐world conditions: manufacturer maximum charge profile (Man‐Max, red) and a commercial EV fast‐charge protocol (Real‐EV, blue). Experimental standard deviation is represented as error bars while model confidence interval, evaluated via jackknife resampling of calibration data, is shown as shaded areas. (c) Simulated minimum potential difference at the negative electrode‐separator interface as a function of charging time, linked to degradation propensity, with higher risk occurring near 0 V. (d) Predicted distribution of the potential difference at the negative electrode along the dimensionless thickness direction (left: current collector; right: separator). Black lines indicate conditions where the potential difference drops below 100 mV across the whole electrode, whereas pink lines depict its distribution at the end of charge, with corresponding times reported accordingly.

Figure 2 compares simulation results with experimental data for two benchmark protocols. The first one (red) consists of constant‐current charging at 4 A (2C), corresponding to the maximum rate recommended by the manufacturer (namely, Man‐Max). The second one (blue), labelled Real‐EV, replicates the dynamic fast‐charge profile implemented by the EV manufacturer Nio for the same battery chemistry, in which the charging current progressively decreases with increasing SoC (see Figure 2a, right axis). Notably, such a Real‐EV protocol is markedly conservative and does not fully exploit the intrinsic rate capability of the cell; nevertheless, it represents a reference, realistic charging scenario for such a battery chemistry. Each protocol is tested experimentally on three pristine cells to ensure statistical robustness, and experimental uncertainty is represented by mean values with error bars indicating standard deviations. After initial electrical characterization to determine the maximum accessible capacity (*Q_M_*, equal to 2.07 ± 0.01 Ah), all cells are cycled within the 20%–80% SoC window using a common discharge procedure (constant current of 2 A (1C) followed by a 20‐minute rest). Differences in battery performance are therefore solely attributable to the distinct charging protocols employed.

Figure 2a,b validates the predictive capability of the calibrated model by comparing simulated macro‐scale indicators (cell potential and temperature) against experimental measurements. Confidence intervals for the model predictions (shaded regions) are estimated using a jackknife resampling approach on calibration data. Figure 2a shows that simulated voltage profiles closely match experimental data, confirming model accuracy even outside calibration conditions. The Man‐Max protocol completes the 20%–80% SoC charge in approximately 18 min, faster than the Real‐EV protocol, which takes around 25 min due to the progressive current reduction beyond approximately 60% SoC. The voltage profile under Real‐EV rises more gently, consistent with lower overpotentials at reduced current densities. Similarly, Figure 2b shows that the model is able to reproduce the measured temperature evolution. The Man‐Max protocol exhibits a slightly higher temperature increase (+7°C, experimental), while the Real‐EV protocol shows an initial temperature rise followed by a reduction as current tapers off, reflecting reduced Joule heating and reaction overpotentials.

The purpose of this study is to design fast‐charging protocols that proactively avoid degradation‐prone regimes, rather than to mechanistically reproduce the onset and evolution of ageing phenomena. For this reason, unlike our previous studies [18], no active modelling of lithium plating is included since the charging protocols under investigation prevent plating initiation altogether. In contrast, a physical model of SEI growth would be valuable, in principle. However, the literature presents studies that lead to markedly different physical interpretations [14], including SEI possessing mixed ion–electron conduction with composition‐dependent electronic properties [60], or SEI growth driven by solvent diffusion [35], neutral lithium transport [61], or electron tunnelling [62] as potential rate‐limiting steps: selecting one mechanism among the many being proposed would be speculative and could introduce bias into the protocol design. For this reason, this study adopts the local potential difference at the negative electrode, Δ*E_n_*, as a conservative operational indicator of degradation risk. Specifically, the time spent with Δ*E_n_* below 100 mV is used as a proxy to quantify plating and SEI propensity. Albeit simplistic, this approach is supported by both modelling and experimental evidence showing that prolonged operation at low Δ*E_n_* accelerates SEI growth [14, 63]. In this context, the present framework does not attempt to resolve the detailed kinetics of SEI formation, but rather to constrain operation within electrochemical conditions that minimize its driving forces.

Having confirmed in Figure 2a,b the accuracy of macro‐scale predictions, the model is subsequently used to resolve and investigate micro‐scale electrochemical behaviors via the analysis of Δ*E_n_*, which is closely linked to degradation phenomena at the graphite electrode as already anticipated. As intercalation proceeds, Δ*E_n_* decreases at the particle surface, promoting SEI growth; under sufficiently severe conditions, negative Δ*E_n_* values may be achieved and indicate the thermodynamic onset of lithium plating. Phase‐separation phenomena in graphite strongly influence these dynamics, especially the premature nucleation of the Stage 1 (LiC_6_) phase at the particle surface, further decreasing Δ*E_n_* [18]. Thus, capturing both local potentials and graphite phase‐separation dynamics is essential for accurately monitoring degradation risks and designing optimized fast‐charge protocols.

Figure 2c specifically examines the evolution of Δ*E_n_* at the negative electrode/separator interface for both protocols. Under Man‐Max (solid line), a monotonic decline is observed, with Δ*E_n_* reaching below 20 mV before achieving 80% SoC. Conversely, Real‐EV (dashed line), through its current modulation strategy, partially relaxes Δ*E_n_*, maintaining a safer margin above the critical lithium plating threshold. To further elucidate spatial degradation dynamics, Figure 2d presents simulated Δ*E_n_* profiles across the graphite electrode thickness at two critical time points: when the electrode first reaches the conservative 100 mV threshold (black lines), associated with accelerated SEI growth [63], and at the end of the charge (pink lines). Both protocols reach the 100 mV threshold at similar times; yet, at charge completion, Real‐EV maintains a smoother, safer Δ*E_n_* gradient compared to Man‐Max, which features more extensive low‐potential regions. This suggests a higher susceptibility to degradation via SEI growth and lithium plating for the Man‐Max protocol, stemming from its sustained combination of low potential and high current.

These results confirm the model's ability to predict critical macro‐ and micro‐scale electrochemical indicators useful to detect degradation under realistic fast‐charge conditions. With this validated predictive framework in place, the attention turns to its use in the development of a new charging protocol aimed at balancing charging speed with long‐term cell stability.

### 2.3 Model‐Informed Fast‐Charge Protocol Design

By dynamically monitoring critical internal variables (e.g., the local potential difference at the negative electrode) and accurately modelling graphite phase separation, the model enables the design of advanced charging strategies that minimize degradation while maintaining high rates without neglecting crucial physics. Our phase‐field modelling framework uniquely captures the nucleation and evolution of phase separation in graphite particles during charging across all tested protocols (Figure S4), a feature inaccessible to solid‐solution models. In fact, neglecting phase separation leads to inaccurate prediction of local potential differences, which are critical for identifying degradation risks such as lithium plating [17, 18, 64].

Based on this principle, the P2D phase‐field model is now employed to design a model‐informed fast‐charging protocol (Mod‐Inf) capable of completing a 20%–80% charge in under 15 min. Notably, this performance target must be achieved without accelerating degradation compared to current commercial protocols. In fact, rapid charging inherently requires elevated current densities, especially at the beginning of the charge, potentially triggering degradation mechanisms at both electrodes. At the positive NMC electrode, rapid delithiation at particle surfaces can induce lattice instability and promote the formation of a detrimental rock‐salt phase if the local lithiation state falls below 30% [65]. At the graphite electrode, transport limitations in electrolyte and solid phases may cause premature nucleation of the Stage 1 phase at particle surfaces, thereby increasing the risk of lithium plating [15, 18].

To address these challenges, we propose an adaptive fast‐charging protocol in which the current is dynamically modulated based on internal electrochemical indicators, specifically the local potential difference at the negative electrode, Δ*E_n_*. It is worth stressing that the generalisable element of the protocol is the control principle itself, not the exact current profile or the specific Δ*E_n_* threshold. Different chemistries and electrode architectures may require their own Δ*E_n_* set‐point to ensure that the negative electrode remains safely above plating conditions. A key advance of this approach is the integration of graphite phase‐field modelling, which improves the reliability of local potential predictions by explicitly resolving graphite staging transitions (Figure S4). This feature is often neglected in earlier models [26, 29, 66, 67]. In the Mod‐Inf protocol, charging begins at a high current of 6.5 A (3.5C) to maximize initial speed. Once the local potential difference at the negative electrode–separator interface (Δ*E_n_*) reaches a predefined threshold of 25 mV, the current is progressively reduced to stabilise Δ*E_n_*. The 25 mV threshold is selected to strike a balance between fast charging and model reliability. Although a lower threshold might allow faster charging, it would approach the accuracy limit of the model. In fact, calibration and validation results indicate a worst‐case RMSE of approximately 20 mV in the 20%–80% SoC window for the cell potential. Setting the threshold at 25 mV maintains a conservative margin above this uncertainty, ensuring that any predicted onset of plating regime would be physically meaningful and not an artefact of model calibration errors.

**FIGURE 3:**
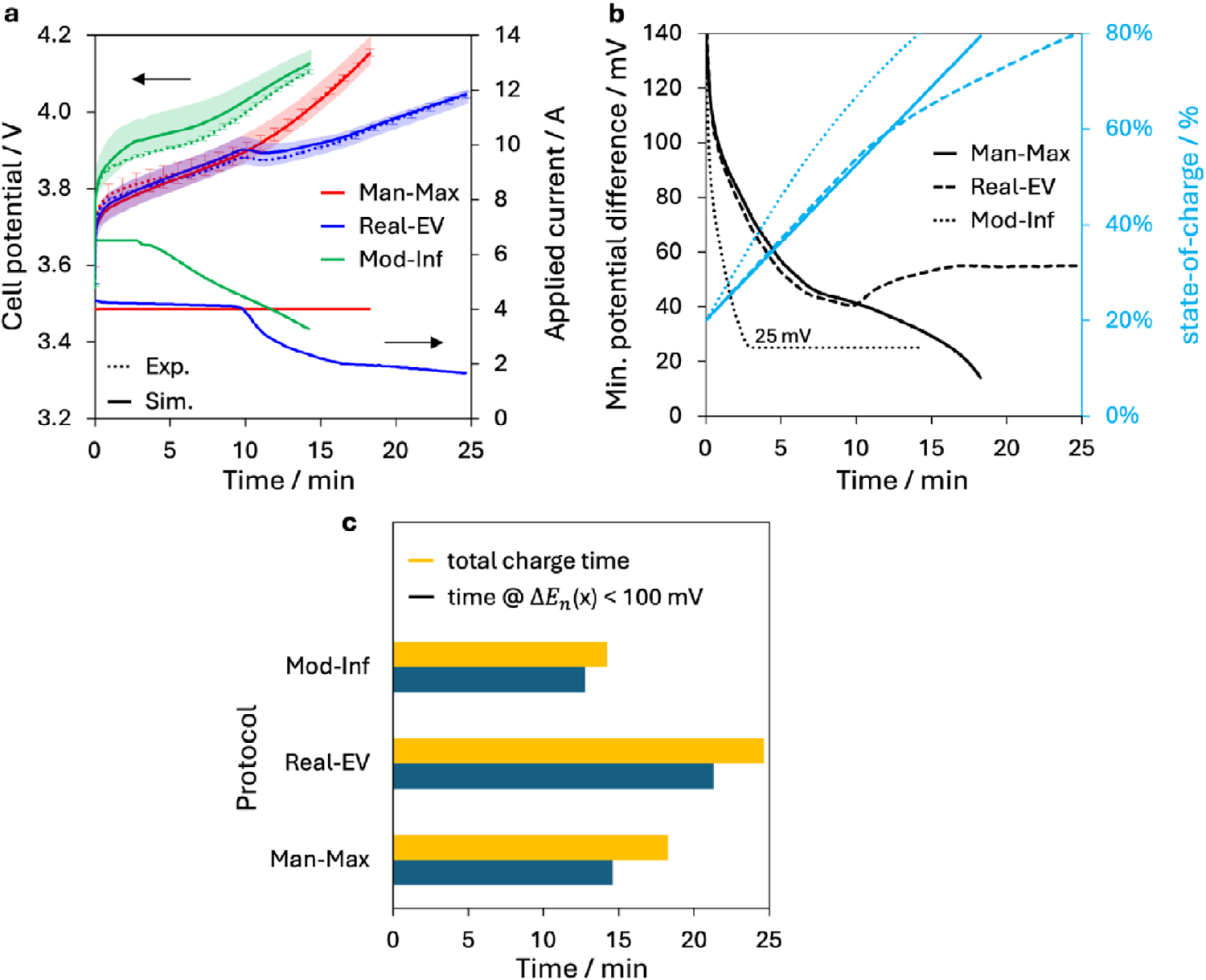
Model‐informed fast‐charge and comparison with the other protocols. (a) Simulated and experimental battery potential for three fast‐charge strategies: manufacturer maximum charge profile (Man‐Max, red), a commercial fast‐charge EV protocol (Real‐EV, blue), and the model‐informed fast‐charge protocol (Mod‐Inf, green). Experimental standard deviation is represented as error bars while model confidence interval, evaluated via jackknife resampling of calibration data, is shown as shaded areas. (b) Simulated evolution of the minimum potential difference at the negative electrode (primary *y‐*axis) and cell state‐of‐charge (secondary *y*‐axis) throughout the charging process. (c) Comparison of total charge time and the time for which the minimum potential difference at the negative electrode (Δ*E_n_*) remains below 100 mV for each protocol.

Figure 3 presents the experimental validation of the Mod‐Inf protocol, benchmarked against the Man‐Max and Real‐EV strategies. Experimental validation involved multiple cell replicates per protocol, with results reported as mean values, standard deviations (error bars), and model uncertainty (shaded regions). Figure 3a demonstrates that the Mod‐Inf protocol successfully achieves charging from 20% to 80% SoC in approximately 14 min, significantly faster than Man‐Max (18 min) and Real‐EV (25 min). This result highlights the model capability to design charging profiles that meet aggressive targets while staying within safe operating limits.

In fact, a detailed examination of Δ*E_n_* during charging is provided in Figure 3b, clearly illustrating differences among protocols. The Real‐EV protocol exhibits increasing Δ*E_n_* in the second half of the charge, reflecting progressive current modulation and system relaxation, whereas Man‐Max shows a steady decline in Δ*E_n_*, indicating increased degradation risk. In contrast, Mod‐Inf consistently maintains Δ*E_n_* near the 25 mV threshold, reflecting its design intent to avoid critical electrochemical conditions throughout the charge. Additionally, the voltage and thermal model predictions closely match experimental measurements (Figure 3a), confirming that Mod‐Inf operates within recommended temperature limits (Figure S3a) and maintains safe lithiation states at the NMC electrode (Figure S3b).

The propensity for degradation at the negative electrode depends not only on the absolute Δ*E_n_* value but also critically on the applied current and exposure time [60, 68]. Indeed, each degradation mechanism follows kinetic laws, making exposure time significant. To quantitatively evaluate this, Figure 3c compares the duration each protocol spends under conditions associated with accelerated degradation (i.e., Δ*E_n_* < 100 mV), as operation below this threshold significantly increases SEI growth and potentially lithium plating [63]. Among the tested strategies, Mod‐Inf achieves the shortest absolute duration in this critical regime, a direct outcome of its adaptive current modulation designed explicitly to minimize exposure to low‐potential conditions while ensuring rapid charging.

The robustness of the Mod‐Inf protocol is evaluated through three targeted sensitivity tests. First, the effect of ambient temperature (5°C–35°C) and initial state‐of‐charge (SoC_in_ = 14%–26%) is assessed while keeping the recharged capacity fixed at 60% of nominal capacity (Figure S17). Across the full range, the 25 mV threshold remains conservative. Δ*E_n_* drops below 0 mV only at low temperatures (<10°C), where pre‐heating would typically be applied [69, 70]. Second, the influence of graphite specific surface area is tested by varying it from the baseline value, calculated assuming spherical particle morphology, to higher values representative of platelet‐like morphologies [19, 51] (Figure S18). No appreciable changes are observed in cell potential, Δ*E_n_* prediction, or time spent below 100 mV, confirming robustness to morphological variability. Third, a comparison of Δ*E_n_* prediction between the single‐radius P2D model and a multi‐particle P2D (MP2D) implementation incorporating a three‐bin particle size distribution shows excellent agreement during charging under the Mod‐Inf protocol (Figure S19), indicating that the design outcome is negligibly sensitive to particle size distribution under the tested conditions. These results confirm that the P2D framework, when equipped with phase‐field physics and validated against MP2D, remains sufficient for protocol design.

While Man‐Max likely induces the highest degradation due to prolonged low Δ*E_n_* under high current, it remains challenging to predict whether Mod‐Inf leads to greater or lesser SEI growth compared to Real‐EV. In fact, the two strategies differ in their current‐time trade‐off: Mod‐Inf applies a higher current over a shorter duration, while Real‐EV operates at a lower current for longer. To clarify this trade‐off and assess the long‐term implications of each protocol on battery ageing, experimental validation through extended cycling is required.

### 2.4 Long‐Term Validation of Fast‐Charge Protocols Through Extended Cycling

While the Mod‐Inf protocol clearly satisfies the target of completing a 20%–80% SoC charge in less than 15 min, its influence on ageing and state‐of‐health (SoH) must be confirmed through direct ageing tests. To this end, the three protocols under investigation (Man‐Max, Real‐EV, and Mod‐Inf) are compared experimentally up to 500 fast‐charge cycles. The same mild discharge protocol (namely, 2 A (1 C) constant current followed by a 20‐minute rest) is adopted in all cases to attribute any difference in ageing to the charging protocols only.

**FIGURE 4:**
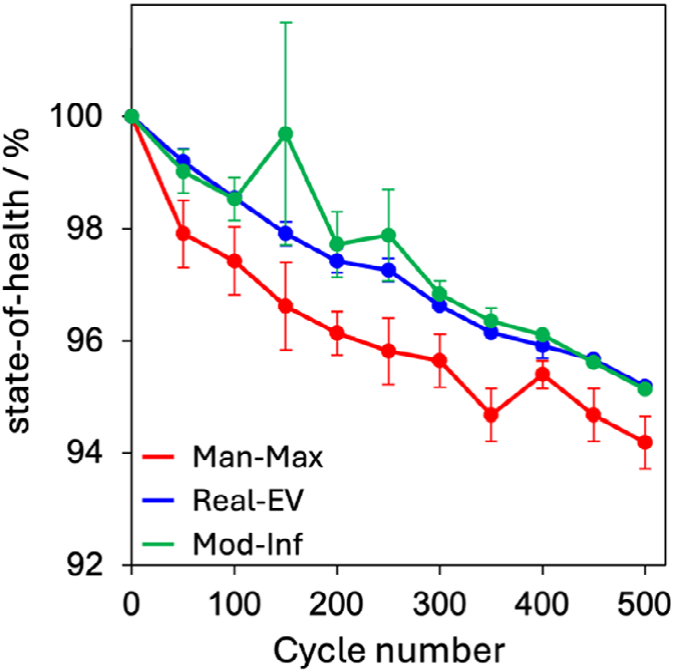
Capacity retention over cycling for different fast‐charge protocols. Evolution of the state‐of‐health (SoH) as a function of cycle number for three fast‐charge protocols applied to the investigated battery: manufacturer maximum charge profile (Man‐Max, red), a commercial fast‐charge EV protocol (Real‐EV, blue), and the model‐informed fast‐charge protocol (Mod‐Inf, green). Each marker represents the SoH evaluated during repetitive performance tests (RPTs) every 50 cycles. Error bars represent the standard deviation calculated from three batteries cycled per protocol, where each battery was charged according to the respective fast‐charge profile and discharged at a constant current of 2 A (1C).

Figure 4 provides a comparative analysis of the long‐term ageing induced by each protocol (see Methods for details on the ageing test procedure). Multiple cells are cycled per protocol to ensure statistical significance, with SoH evaluated periodically through repeated performance tests (RPTs) conducted every 50 cycles. The average evolution and standard deviation of SoH are reported in Figure 4 for the three protocols, clearly highlighting their differences in terms of degradation behavior.

Specifically, the Man‐Max protocol, characterized by a constant 4 A charging current, resulted in the highest degradation rate, with SoH declining to approximately 94% after 500 cycles. A pronounced initial capacity drop within the first 50 cycles is observed, attributed to prolonged exposure at conditions favorable for degradation, particularly SEI growth, due to localized low potential difference at the negative electrode as shown in Figure 3b. In contrast, both the Real‐EV and Mod‐Inf protocols demonstrated less degradation, retaining approximately 95% SoH at the conclusion of cycling. Although the numerical disparity (94% vs. 95%) may appear minor, this difference is statistically significant, clearly exceeding the experimental measurement uncertainty, which is generally quite narrow. In fact, only for the Mod‐Inf protocol at 150 cycles there appears to be a wider standard deviation in SoH, which is attributable to a single cell which likely experienced a slightly higher temperature during that specific RPT, resulting in a transient capacity increase.

Interestingly, despite its notably shorter charge duration (14 min for Mod‐Inf vs. 25 min for Real‐EV) and comparatively higher current peak (3.50C vs. 2.15C), the Mod‐Inf protocol displayed comparable long‐term ageing to the Real‐EV strategy. This highlights the critical role that exposure time and applied current play alongside electrode potential in determining battery degradation, as discussed in the previous section. In fact, while the Mod‐Inf protocol operates at higher currents, it significantly reduces the duration spent in the SEI‐promoting regime (where current is progressively reduced), thereby potentially offsetting kinetic penalties associated with higher rates. Further implications on SEI kinetics will be the subject of future dedicated modelling studies. Importantly, the absence of any signs of accelerated ageing over 500 cycles demonstrates the long‐term viability of the proposed model‐informed charging protocol. While the improvements reported here may be regarded as incremental in absolute terms, which is expected given the maturity of the commercial cell used and the focus on protocol optimization rather than material design, the significance of the result lies in demonstrating that physics‐based modelling, when equipped with a rigorous description of battery physics, can successfully achieve fast charging with concurrent long‐term durability. Most existing modelling studies rely on simplified solid‐solution or empirical models that do not capture the true internal dynamics of commercial graphitic electrodes. By integrating phase‐field physics into a fully calibrated P2D framework, these results show that protocol performance can still be improved without altering cell chemistry or structure. These findings highlight that even in well‐engineered commercial systems, additional performance can be unlocked through accurate prediction of internal electrochemical states.

To contextualize these figures in the EV scenario, the battery experiences only 5% capacity loss after 500 repeated fast‐charging cycles within 20%–80% SoC, which corresponds to an effective usage of over 150 000 km for an EV with approximately 500 km driving full range. This underscores the practical relevance of the Mod‐Inf protocol, highlighting its capability to deliver sustained fast‐charging performance without compromising battery integrity over typical EV lifespans.

These results confirm that the selected commercial cell exhibits limited capacity fade even under sustained high‐rate cycling. The modest degradation observed for both the Man‐Max and Real‐EV protocols reflects the high quality and robustness of the cell design, making any further improvement increasingly challenging. Yet, physics‐based control of internal states can unlock additional performance gains without changes in cell design or chemistry.

### 2.5 System‐Level Implications of Fast‐Charge Protocols

**FIGURE 5:**
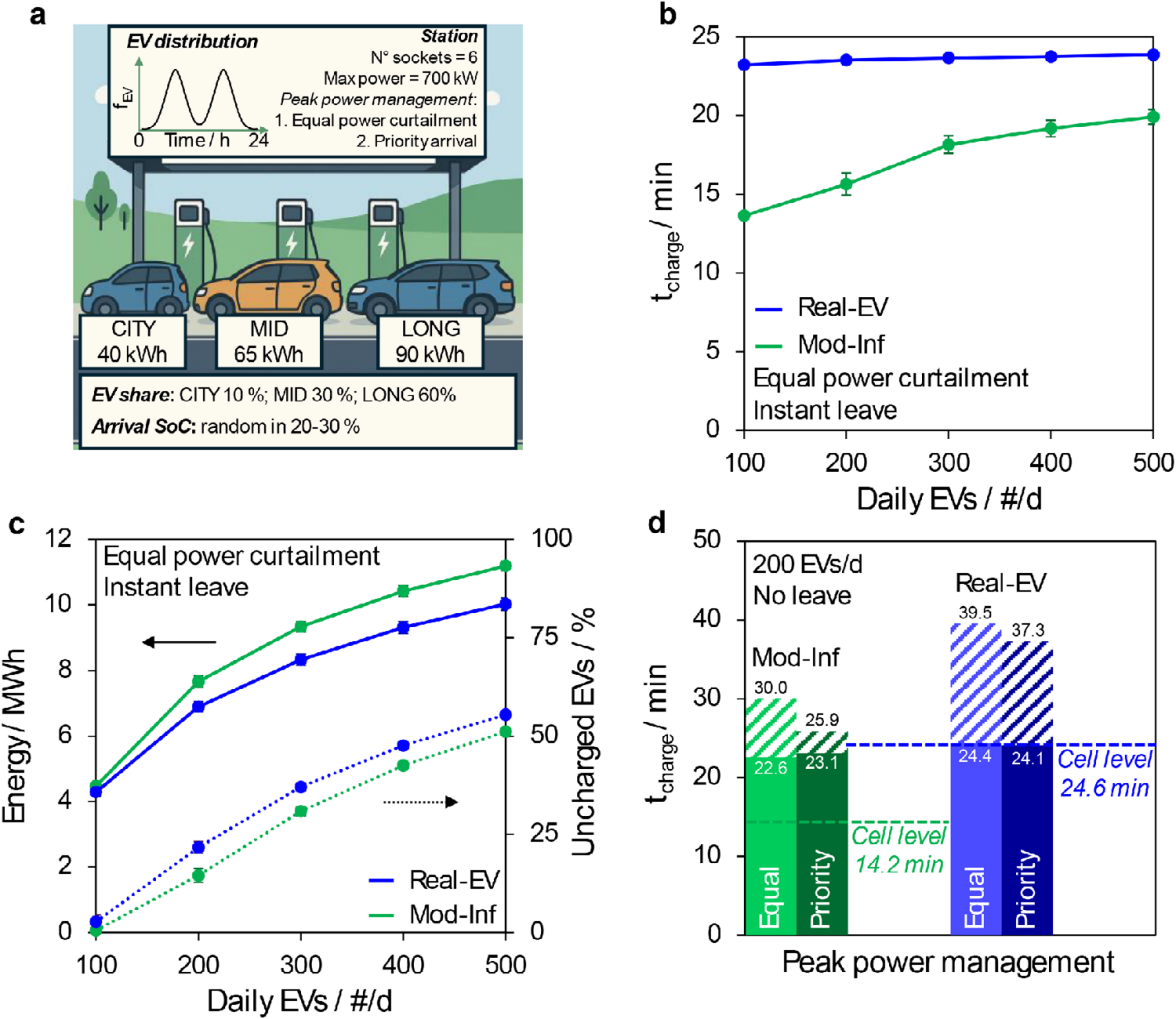
System‐level comparison of Mod‐Inf and Real‐EV fast‐charge protocols via Monte Carlo analysis. (a) Schematics of the simulated charging station and electric vehicle (EV) specifications. (b) Comparison of the total charging time as a function of daily EV throughput for the model‐informed fast‐charge protocol (Mod‐Inf, green) and the commercial EV fast‐charge protocol (Real‐EV, blue), under instant leave (no waiting if sockets are occupied) and equal power curtailment (for peak management). (c) Comparison of energy delivered (solid lines, left *y*‐axis) and percentage of uncharged vehicles (dotted lines, right *y*‐axis) for the two protocols under the same conditions. (d) Comparison of total charging time at 200 EVs/day assuming no leave if sockets are occupied, of the two station management strategies: equal power curtailment (light colors) and priority‐based charge by arrival time (dark colors). Solid columns report the actual charge time, while patterned columns indicate queue time for Mod‐Inf and Real‐EV fast‐charge protocols. Dashed lines represent cell‐level charging times. Error bars (where applicable) represent the standard deviation from 100 Monte Carlo simulations.

While at the cell level, the model‐informed fast‐charge protocol proved to yield comparable long‐term ageing to the Real‐EV protocol, while saving more than 10 min of charge time, its broader implications on the fast‐charging infrastructure need to be assessed. To evaluate station‐level performance under realistic conditions and for different charging protocols, a dedicated framework was developed. This multi‐scenario approach leverages Monte Carlo simulations, in which key parameters are randomly sampled to capture the stochastic nature of real‐world operation. Let us assume to use the investigated Li‐ion cell in EV battery packs and simulate a typical day at a charging station, with 6 sockets and a maximum power of 700 kW, located on a European highway (Figure 5a). For the vehicles approaching the station, having a random SoC between 20% and 30%, charging up to 80% SoC is simulated by adopting either the Mod‐Inf or the Real‐EV charging protocol. The daily arrival distribution and the share of EV classes with different battery packs (40 kWh, 65 kWh, and 90 kWh) are summarized in Figure 5a. Further details of the Monte Carlo simulation are reported in the Methods section.

Figure 5b compares, for the two protocols, the median charging time as a function of the number of vehicles approaching the station per day, which is a proxy of EV market penetration. In this specific scenario, if the aggregate power demand exceeds the station capacity, an equal power curtailment is adopted for all EVs in charge; furthermore, if no socket is available, any new arrival is assumed to immediately leave the station (we will relax these assumptions later in this section). Numerical results in Figure 5b show that, at all levels of daily demand, the Mod‐Inf protocol consistently yields shorter median charging times than the Real‐EV protocol. This advantage is observed across all three vehicle classes at fixed daily demand, as shown in Figure S20a, and is more pronounced under moderate traffic conditions. For instance, at 100 EVs per day, the median charging time up to 80% SoC is less than 14 min for the Mod‐Inf protocol, compared to ca. 23 min for Real‐EV. As station utilization increases, the median charging time of the Real‐EV protocol remains comparatively stable. Conversely, the Mod‐Inf protocol shows a more pronounced increase in charging time. In fact, due to its more aggressive current profile, the Mod‐Inf protocol approaches the station power cap more frequently as the number of EVs grows. In these cases, repeated power curtailment events extend individual charging sessions, resulting in a steeper rise in the median charging time compared to the inherently less demanding Real‐EV protocol. This effect is not intrinsic to the protocol itself but, rather, it emerges from an interplay with infrastructure constraints, underscoring that cell‐level optimization must be matched by adequate power provisioning at the station level (see Figure S20b,c). In any case, Figure 5b indicates that, from a user perspective, the Mod‐Inf fast‐charge protocol is preferable to the Real‐EV one.

The benefits introduced by the Mod‐Inf protocol translate also to the operating company of the charging station, whose interests are in maximizing the total energy delivered per day and in minimizing the fraction of uncharged EVs which left the station. As shown in Figure 5c, the Mod‐Inf protocol surpasses the Real‐EV protocol in both performance indicators regardless the number of EVs approaching the station. The shorter charging time ensured by the Mod‐Inf protocol frees the sockets more quickly, making them available to accommodate and serve more EVs per day, thus ultimately delivering more energy and promoting station utilization at maximum capacity (Figure S20b). The high fraction (i.e., >50%) of unserved vehicles at 500 EVs/day indicates that the station is significantly undersized beyond this condition, making the analysis irrelevant for higher penetration scenarios. The differences between the Mod‐Inf and Real‐EV charging protocols in both total energy delivered (ca. +10% on average) and fraction of uncharged vehicles (ca. −5% on average), both of which clearly increase with the traffic load, are greater than the standard deviations (represented as error bars) due to the randomness of Monte Carlo simulation, thus revealing statistical significance of the results.

All these results point at the advantages, from both user and operator perspectives, introduced by the Mod‐Inf fast‐charge compared to current charging protocols. Nevertheless, it is worth investigating if such benefits are robust even for different management strategies and user behaviors, thus relaxing some of the assumptions used above. Figure 5d compares the two charging protocols under the opposite extreme scenario of ‘instant leave’ user behavior, that is, the ‘no leave’ behavior; in other words, if no socket is available, the EV arrived at the station remains indefinitely in queue until a free socket is made accessible. In addition to the equal curtailment management strategy of power peak demand, a priority‐based strategy is also explored in Figure 5d: in case of power demand by EVs on charge exceeding the station cap, electric power is curtailed only at the sockets of later arrivals at the station.

For the representative condition of 200 EVs per day, Figure 5d shows that the median time of an EV at the station raises to 25.9 min (priority‐based peak management) or 30 min (equal‐curtailment peak management) for the Mod‐Inf protocol; such figures comprise from 2 to 7 min of queuing time, while the effective charge time at the socket is in the order of 23 min (solid green bars in Figure 5d). These total charging times are significantly larger than the 20%–80% SoC charging time enabled at the cell level by the Mod‐Inf protocol (green dashed line in Figure 5d). The Real‐EV protocol (blue bars) suffers from longer times at the station too, even beyond 37 min in total, mostly due to excessive queuing (>13 min, blue striped bars). In fact, the low‐power Real‐EV protocol rarely hits the maximum station power, thus rarely necessitating power curtailment and so ensuring a charging time at the socket comparable to the 20%–80% SoC cell‐level charging time (blue dashed line); on the other hand, its comparatively longer duration keeps the sockets occupied, thus creating queues of vehicles at the station waiting for the next available socket. In any case, Figure 5d highlights two key results: (i) the priority‐based peak management, which allocates power preferentially to earlier arrivals, is superior no matter the charging protocol, favoring especially intrinsically faster protocols; (ii) even in such extreme condition, the Mod‐Inf protocol completes the charge up to 80% SoC at the station in a total time comparable to that of the Real‐EV protocol at cell level. In a prospective future scenario representing next‐generation charging infrastructure, where station capacity is assumed to double to over 1 MW, as shown in Figure S20d, the Mod‐Inf protocol completes charging in just 13.3 min regardless of power management strategy, with no queueing or curtailment. In contrast, the Real‐EV protocol gains limited benefit from this upgrade.

The analysis presented in this section demonstrates that the Mod‐Inf protocol is already applicable in the current charging infrastructure, providing benefits for both EV drivers and station managers. However, to really unlock its potential, charging stations must be redesigned to sustain higher peak powers. While these indications result from the model‐informed fast‐charge protocol conceived in this study, we believe that these considerations should generally apply to all the fast‐charge protocols that may be deployed in the coming years.

## 3 Discussion

This study presented a comprehensive cell‐to‐system validation of a model‐informed fast‐charge protocol that merges rapid charging with long‐term battery health. Using a non‐equilibrium phase‐field framework calibrated through detailed experimental characterization, the proposed strategy enables 20%–80% SoC charging in under 15 min while maintaining less than 5% capacity loss over 500 cycles. The protocol outperforms both manufacturer‐recommended and real‐world EV charging profiles, not only at the cell level but also when integrated into system‐level simulations of highway fast‐charging infrastructure. These findings demonstrate that physics‐based, degradation‐aware optimization can deliver substantial performance gains without compromising battery longevity or infrastructure compatibility, offering a viable path toward fast, safe, and scalable charging for next‐generation electric vehicles without dramatic changes in battery chemistry or cell design.

Our results advance the state of the art in model‐informed charging strategies by shifting from empirical or data‐driven optimization to predictive, physics‐based control of internal battery states. A key distinction from previous approaches lies in the physical description of graphite lithiation dynamics. In contrast to conventional fast‐charging studies that describe graphite as a solid‐solution material and rely on elevated temperatures to accelerate kinetics, our model explicitly incorporates the thermodynamics of phase separation. Phase separation in graphite is a fundamental material property; a solid–solution framework, regardless of calibration effort, cannot capture critical features such as spinodal decomposition and voltage plateaus, which govern local potential evolution and plating risk. By using a phase‐field formulation rigorously calibrated for commercial‐grade electrodes, the present modelling approach enables accurate tracking of the local potential difference at the graphite–electrolyte interface, which is dynamically maintained above a safety threshold, set at 25 mV in this specific study. This allows the charging current to be dynamically modulated to avoid entry into degradation‐prone regimes, enabling fast charging with controlled degradation.

Notwithstanding the statistical significance of the current results, several opportunities exist to further strengthen the model and extend its generalizability. While the present study demonstrates excellent capacity retention and minimal degradation over 500 cycles, direct diagnostics of degradation modes, such as post‐mortem scanning electron microscopy and impedance spectroscopy on degraded cells, were beyond the scope of this study. Integrating these characterization techniques into future analyses would provide essential evidence to confirm the suppression of lithium plating and better elucidate underlying degradation mechanisms, particularly at particle and electrode interfaces.

The present framework does not include an explicit mechanistic description of SEI growth kinetics and therefore does not aim at quantitative lifetime extrapolation beyond the tested conditions. The conclusions drawn here are grounded on experimental validation within 500 consecutive fast‐charge cycles. Extrapolation beyond this validated window should be approached with care, although in real‐world usage an EV would unlikely undergo more than 500 fast‐charge cycles during its lifetime. Nevertheless, quantitative descriptions of SEI growth dynamics under high‐rate cycling conditions should be integrated within the phase‐field framework in future developments to enhance long‐term predictive capability.

Broader implementation of this framework will require extension beyond the single‐cell level to capture dynamics at the module and pack scales. In this context, integration with distributed thermal descriptions represents a natural progression of the present framework. It is well established that cell‐to‐cell variability, internal thermal gradients, and local cooling constraints can introduce non‐uniformities that affect charging behavior in real‐world battery packs. These effects do not undermine the control principles developed in this study, but they must be explicitly addressed through the inclusion of distributed thermal models and pack‐level coupling.

While the specific fast‐charge protocol developed in this work is tailored to the tested NMC/graphite 18650 cell and validated under nominal conditions at 20°C, the methodology itself is broadly applicable to other chemistries, formats, and thermal environments. Although the sensitivity analysis confirms robustness within a moderate temperature window, low‐temperature fast charging remains a critical operating condition due to increased plating propensity and transport limitations. Dedicated protocol adaptation and extended experimental validation under sub‐ambient conditions are therefore required to fully assess behavior during cold‐start scenarios.

At present, experimental validation has been conducted only for the investigated NMC/graphite system. Extension to alternative electrode materials would require chemistry‐specific equilibrium potentials, transport parameters, and degradation thresholds to be identified through dedicated characterization and calibration. Once a new system is experimentally characterized and the relevant electrochemical thresholds are identified, including a suitable Δ*E_n_* threshold for plating control, the same model‐based workflow can be applied to design robust fast‐charging strategies. In parallel, longer‐term validations under diverse operational conditions including variable temperatures, broader charging rate distributions, and realistic EV usage profiles, are essential to assess robustness and scalability. The extension of this modelling approach to alternative battery chemistries, such as silicon‐graphite composite, high‐nickel, and lithium iron phosphate (LFP) systems, will further test the generalizability of our framework and protocols. Such investigations would enable the widespread application of degradation‐aware charging strategies across both consolidated and emerging battery technologies, facilitating broader industrial and commercial impacts.

Taken together, the results support the broader vision of digital twin frameworks for battery systems, in which multiscale physics‐based models inform real‐time operational decisions spanning the cell, vehicle, and charging infrastructure levels. By validating the proposed model‐informed protocol across this complete system scale, that is, from internal electrochemical phenomena to public charging station performance, this study lays a robust foundation for deploying next‐generation fast‐charging solutions that are not only high‐performing and degradation‐aware but also fully compatible with existing and future infrastructure.

## 4 Methods

### 4.1 Tested Batteries

Samsung INR18650‐20R commercial batteries (graphite/nickel manganese cobalt oxide) were employed in this study in all tests. The batteries feature 2 Ah nominal capacity (*Q_nom_*, based on which C‐rate is defined), a nominal potential of 3.6 V, and an operative potential range from 2.5 to 4.2 V. The allowed temperature range during charge was 0°C to 50°C while −20°C to 75°C during discharge. Fast charge current applicable from the manufacturer's datasheet was a constant current of 4 A (2C) until 4.2 V potential cut‐off was reached.

### 4.2 Electrical Setup

Batteries were cycled using a Chroma 69225‐100‐4 battery cycler, whose specifications are reported in Figure S5. The batteries were electrically connected using a battery holder with a two‐point contact connection. The batteries were placed inside an FDM300 climatic chamber (Figure S5) which was maintained at 20°C during all electrical tests. Each tested battery was equipped with a K‐type temperature probe to monitor temperature variations during operation, while an additional K‐type probe monitored and regulated the internal temperature of the climatic chamber. The experiments featuring charge/discharge and ageing protocols were conducted at the DESTEC Battery Lab at the University of Pisa within a climate‐controlled room. Data acquisition was performed using Chroma proprietary software, and the raw data are provided as described in the Data Availability section.

Electrochemical impedance spectroscopy (EIS) tests were performed with an Autolab PGSTAT302N potentiostat/galvanostat coupled to a frequency response analyzer FRA32M, both produced by Metrohm (Figure S6). EIS data were acquired in galvanostatic mode. The batteries were electrically connected using a battery holder with a two‐point contact connection. EIS tests were conducted at the DICCA electrical laboratory at the University of Genova within a climate‐controlled room without a climate chamber. Data acquisition was performed using NOVA Autolab proprietary software, and the raw data are provided as described in the Data Availability section.

### 4.3 Charge/Discharge Protocols

To ensure high‐quality and reproducible data for model calibration and analysis, different constant current charge and discharge protocols were designed and applied on three battery cells to assess repeatability. These protocols defined the electrochemical state of the battery (i.e., the electrode's state of lithiation at 0% and 100% SoC) and enabled a systematic evaluation of battery performance indicators (i.e., cell potential, capacity, and temperature) under controlled conditions.

#### 4.3.1 Pre‐Discharge (PREDCH)

It was applied before the initial charge cycle to establish a well‐defined starting condition. The battery was discharged at 0.4 A (0.2C) until the voltage reached 2.5 V. The current was then stopped, and the battery was left at open circuit for 1 h.

#### 4.3.2 Standard Charge (STDCHA)

The 100% state‐of‐charge was established and restored a fully charged condition after each experiment. The battery was charged at 1 A (0.5C) until the voltage reached 4.2 V. The cell potential was maintained at 4.2 V until the current dropped to 10 mA (1/200C). The charge was then stopped, and the battery was left at open circuit for 1 h.

#### 4.3.3 Reference Discharge (REFDCH)

The maximum accessible capacity (*Q_M_*) was determined and defined the 0% SoC. The battery was discharged at 0.2 A (0.1C) until 2.5 V was reached, followed by a constant voltage (CV) phase at 2.5 V until the current drops to 10 mA (1/200C). The discharge was then stopped, and the battery was left at open circuit for 1 h. The maximum accessible capacity (*Q_M_*) was determined as the total capacity delivered during the CC and CV phases. *Q_M_* was also used to define the battery state‐of‐charge as *SoC*  =  *Q*/*Q_M_* , where *Q* is the capacity discharged (charged) during operation.

#### 4.3.4 Discharge–Charge Cycling (CYCLEX‐XA)

Battery performance was evaluated at different currents, as shown in Figure 1a. The protocol starts from a fully charged battery following the STDCHA protocol and 2 h rest at open circuit to ensure thermal and electrochemical equilibration. The cell was discharged at a predefined constant current (X‐XA) (namely, 0.2, 0.5, 1, 2, 4 A) until 2.5 V was reached. The voltage was kept constant at 2.5 V until *Q_M_* was extracted (so that 0% SoC was attained). After resting for 1 h, the battery was charged at constant current X‐XA until 4.2 V was reached. Charging continued at a constant 4.2 V until the total charged capacity equals *Q_M_* (so that 100% SoC was fully restored), followed by 1 h rest.

#### 4.3.5 Discharge Galvanostatic Intermittent Titration Technique (GITTDCH)

The complete open‐circuit potential (OCP) profile of the battery was evaluated from 0% to 100% SoC (Figure S13). This protocol followed a standard GITT approach, where discharge phases were interspersed with rest phases. After STDCHA, the battery was discharged at a constant current of 0.4 A (0.2C) for 15 min, such that the capacity discharged during each pulse was 5% of *Q_nom_*, where *Q_nom_* is the nominal capacity specified in the manufacturer datasheet (2 Ah). After each pulse, the battery was left at open circuit for 1 h, and the cell potential at complete equilibration was recorded. This procedure was repeated an additional 19 times until *Q_nom_* was extracted. Since *Q_M_*  >  *Q_nom_*, the OCP at 0% SoC was evaluated by continuing cell discharge with the REFDCH protocol until the maximum accessible capacity was extracted; then, after 1 h rest, the equilibrium potential was recorded.

For galvanostatic cycling tests, the root mean square error (RMSE) was calculated as:

(1) $$RMSE=\sqrt{\sum_{i=1}^{N}\frac{{\left(X_{sim,i}-X_{exp,i}\right)}^{2}}{N}}\;$$

where *N* is the number of datapoints collected during the test, *X* is either battery voltage or temperature, and subscripts *sim* and *exp* denote model predictions and experimental data, respectively.

### 4.4 Electrochemical Impedance Spectroscopy Experiments

Electrochemical impedance spectroscopy (EIS) data were collected on pristine batteries whose maximum accessible capacity *Q_M_* was assessed from the sequence of PREDCH, STDCHA, and REFDCH protocols as described above. Starting from 0% SoC, after 2 h equilibration at open circuit, the cell was charged at 20% SoC with a constant current of 0.2 A (0.1C). After 1 h rest, three consecutive impedance spectra were acquired via sinusoidal perturbation in galvanostatic mode with 10 mA current amplitude within 10^6^–5 × 10^−2^ Hz, recording 10 frequency points per decade. The repetition of three consecutive measurements assessed the consistency of the EIS response, while the chosen current amplitude ensured the linearity condition. After 1 h rest at open circuit, the SoC was incremented by 15% via a constant current step of 0.2 A, then the rest, EIS measurements, and SoC increments were repeated until 80% SoC. Thus, EIS spectra were collected at 20%, 35%, 50%, 65%, and 80% SoC, as shown in Figure 1b. As a separate test, a single impedance measurement with the same characteristics described above was conducted on the test bench without the cell to measure and decouple the impedance contribution of the rig and connecting cables from that of the battery.

For EIS validation, the relative impedance error Was Calculated as:​

(2) $$e\;\left(f\right)=\frac{\left|Z_{sim}\left(f\right)\;-\;Z_{exp}\left(f\right)\right|}{\left|Z_{exp}\left(f\right)\right|}\;$$

where *Z_sim_* and *Z_exp_* were the simulated and experimental impedance at frequency *f*, respectively. To provide a single representative metric, the mean relative error ${\bar{e}}_{tf}$ was obtained by averaging *e*(*f*) across the frequency range.

### 4.5 Ageing Experiments

Nine cells were cycled, strictly within 20%–80% SoC, using different fast‐charge protocols to assess the evolution of the battery state‐of‐health (SoH) in 500 consecutive cycles. The ageing tests were conducted in a climate chamber maintained at a constant temperature of 20°C throughout all tests. Each ageing procedure consisted of a charge–discharge sequence, interspersed by repetitive performance tests (RPTs) every 50 cycles, where the discharge conditions were the same for each ageing procedure, while the fast‐charge protocol varied.

The battery state‐of‐health at cycle number *i* (*SoH_i_*) was defined as the ratio between the maximum accessible capacity evaluated at the *i*‐th cycle (*Q*_*M*,*i*_) and the maximum accessible capacity of the pristine battery (*Q*_*M*,0_), both evaluated via the sequence of PREDCH, STDCHA, and REFDCH protocols, whose combination represented the RPTs performed every 50 cycles. The corresponding maximum capacity *Q*_*M*,*i*_ was also used to define the battery SoC for the following block of 50 cycles. Following the RPT, the battery was set at 20% SoC with a constant current of 1 A (0.5C) and left at open circuit for 1 h, after which the ageing procedure started/continued from the fast‐charge phase.

The ageing procedure cycled the battery within 20%–80% SoC by adopting the same discharge conditions (constant current of 2 A (1C) followed by 20 min rest), while the fast‐charge protocol differed. Three fast‐charge protocols were tested in different series of ageing experiments, namely: manufacturer maximum current (Man‐Max); reconstructed commercial fast‐charge protocol adopted by Nio EV cars (Real‐EV); model‐informed fast‐charge protocol (Mod‐Inf). These three protocols were followed by 20 min rest at open circuit before discharging the cell and repeating the cycle. While Man‐Max featured a constant current charge phase (2 A), both Real‐EV and Mod‐Inf protocols used a current profile that was modulated over time (i.e., as the SoC changed) as shown in Figures 2a and 3a. Within each block of 50 cycles, the same capacity, equal to *Q_i_*  =  0.6  ×  *Q*_*M*,*i*_ (where *Q*_*M*,*i*_ is the maximum capacity measured in the preceding RPT), was strictly circulated in both charge and discharge phases: in none of the tests performed did the cell potential exceed the manufacturer's limits (namely, 2.5 V during discharge and 4.2 V during charge).

After 250 cycles, one cell per protocol group was removed for a dedicated mid‐life teardown and degradation analysis, the results of which would be reported in future investigations. The remaining cells continued cycling to complete 500 cycles.

### 4.6 Battery Teardown and Characterization Analysis

Teardown and characterization analysis were conducted on three pristine Samsung INR18650‐20R cylindrical cells to obtain statistically relevant parameters.

The following methodology was applied to each battery (Figures S7–S11). After the determination of *Q_M_*, the battery was further discharged at 0.2 A (0.1C) until it reached a voltage limit of 2 mV, here denoted as nominally 0 V (Figure S1). During this phase, the discharged capacity was recorded and summed to *Q_M_*, allowing the total discharged capacity down to 0 V ($Q_{M}^{0V}$) to be determined. The cell was weighed, and its external geometrical dimensions were measured using a caliper before teardown. The cell was then transferred into an MBraun LABstar glovebox environment for disassembly and jelly roll extraction, following best practices for cell characterization [71, 72]. After disassembly, each component of the jelly roll was weighed, and its dimensions were measured. The positive and negative electrodes consisted of a double‐coated current collector with active material layers. For each electrode composite, three sections were collected from the left, central, and right regions, as shown in Figures S7–S10, and analyzed using a statistical approach. FEI Quanta FEG 450 scanning electron microscopy (SEM) was used to acquire 2D top‐view images of battery electrodes and processed using ImageJ software to extract the particle size distribution (Figure S11). The chemical composition of the electrodes was determined via inductively coupled plasma optical emission spectrometry (ICP‐OES) analysis conducted with a Thermo Fisher Scientific iCAP 7400DUO spectrometer, while carbon‐based components in both the negative and positive electrodes were characterized via thermogravimetric analysis (TGA) conducted with a TA Instruments Q500 analyzer. An extended version of the characterization analysis, including the full set of measured parameters and additional experimental details, is provided in the Supporting Information, along with schematics of procedures followed to extract model parameters.

### 4.7 Pseudo‐2‐Dimensional Homogenized Phase‐Field Model

The phase‐field model employed in this study extended the well‐established pseudo‐2‐dimensional (P2D) framework, originally introduced by Newman and colleagues [73, 74, 75], and already presented in previous works from our group [18, 51]. Rooted in porous electrode theory, the P2D model provided a mesoscopic, homogenized description of mass and charge conservation in both the solid and liquid phases within a single repeating battery unit [76]. The through‐thickness coordinate (*x*) from negative to positive current collector was treated as a 1D domain, coupled with Li transport along the radial coordinate (*y*) within active material particles, introducing a pseudo‐dimension. The model represented battery components as a continuum, incorporating microstructural information and particle properties through effective kinetic and transport parameters. Across the through‐thickness direction, the model solved for mass and charge conservation of Li ions, determining their reduced electrochemical potential (${\tilde{\mu }}_{+}^{*}$) and concentration (*c*) in the electrolyte phase based on concentrated solution theory [77, 78]. The coupling between the 1D through‐thickness domain and the pseudo‐1D radial direction was regulated by the charge‐transport intercalation reaction, modelled according to Butler–Volmer kinetics. Within each electrode, electron transport in the electron‐conductive phase (*el*) followed Ohm's law via a gradient in reduced electrochemical potential of electrons (${\tilde{\mu }}_{e}^{*}$). To describe Li transport in the active materials, the P2D framework was extended with a phase‐field approach, based on the mathematical formulation introduced by Bazant and co‐workers [44, 79, 80]. This framework could be generalized to non‐phase‐separating materials as well [51, 57, 81]. Specifically, intercalated Li concentration in active material particles (*c_s_*) was solved according to a microscopic mass balance along the particle radius [39, 77, 81]. The Li flux follows non‐equilibrium thermodynamics [18, 46, 51, 55, 57, 80, 81], where mass transport was driven by the gradient of dimensionless chemical potential ($\nabla {\tilde{\mu }}_{s}$) instead of the concentration gradient (∇*c_s_*). The chemical potential was derived from the Gibbs free energy functional (*G*), which depended on local intercalated Li concentration and, for phase‐separating materials as graphite, included a non‐local term proportional to ∇^2^ *c_s_* to capture uphill diffusion and phase separation in a Cahn–Hilliard fashion [47]. The open‐circuit potential (*E_eq_*) of both electrodes was defined using Nernst's law [79, 81]. The electrochemical model was coupled with a 0D thermal model to account for cell temperature variations during operation, following the well‐established framework introduced by Bernardi et al. [82, 83, 84, 85, 86], accounting for reversible and irreversible heat contributions. Notably, the lumped approach was chosen as this study targets commercial 18 650 cells to minimize heterogeneous thermal gradients during high‐rate operation that would otherwise introduce unnecessary modelling complexity at this scale. Under the present experimental conditions, the assumption of spatially uniform temperature was justified by the relatively small cell dimensions and controlled thermal environment. However, in larger battery modules and packs, non‐uniform cooling and current distribution might generate significant temperature gradients that were not captured by the present formulation. Extension toward distributed thermal models would therefore be required for detailed pack‐level thermal and electrical prediction. The model was implemented in COMSOL Multiphysics V6.2 [87], where a combination of time‐dependent simulations and linear perturbation studies was conducted to reproduce charge/discharge and EIS tests, respectively. To assess the robustness of the charging protocol, we extended the P2D framework by incorporating the experimentally determined particle size distribution, derived from SEM image analysis (Figure S11), thereby obtaining a multiparticle P2D (MP2D) formulation implemented as a discrete set of particle radii with corresponding volume fractions. The three‐bin discretization was selected so that the volume‐weighted effective particle radius matched the single effective radius used in the original P2D model (Figure S19). The detailed mathematical formulation, along with parameter definitions and boundary conditions, is provided in the Supporting Information.

### 4.8 System‐Level Monte Carlo Analysis

To assess the performance of the model‐informed and commercial EV fast‐charge protocols on charging stations under different user preferences and station management strategies, a Monte Carlo simulation framework was developed [59] and implemented in MATLAB R2024b. The simulation covered a 24‐hour operational cycle and was repeated for 100 independent realizations to account for stochastic variability in EV arrivals and states of charge (SoC). Each realization begins by generating EV arrival times based on a discrete probability distribution derived from real‐world station usage data [88]​. Vehicles were randomly assigned to one of three classes (city, medium, long range) based on predefined probabilities, each associated with different battery capacities and pack configurations. Upon arrival, each EV was assigned an initial SoC randomly distributed between 20% and 30%. At every simulation second, EVs arriving were queued if no sockets were available. The charging station included 6 sockets and a maximum power of 700 kW. If the total requested power exceeded this limit, two socket management strategies were available: (i) Equal Power Curtailment, in which all EVs in charge reduce their charging power proportionally, and (ii) Priority Arrival, in which lower‐priority EVs (i.e., those arrived later) were selectively curtailed to maintain the station power limit, favoring earlier arrivals. SoC progression for each EV was updated every second, scaling the single‐cell charging power by the number of cells in the EV pack. Vehicles remain in queue until either (i) a socket becomes available, or (ii) a maximum queue time is exceeded, after which they leave uncharged. Vehicles completed charging upon reaching a target SoC of 80%. Output performance indicators, including the number of vehicles charged and uncharged, total station energy delivered, and median queue and charge times, were aggregated across all realizations to calculate means and standard deviations. To isolate the impact of protocol dynamics from external logistical factors, simplifications such as instantaneous plug‐in/out and negligible socket cooling times were assumed. Consequently, the reported charging times represented best‐case operational conditions. In practical deployments, additional delays associated with communication, scheduling, or thermal stabilization would introduce time offsets. These offsets, however, would be largely independent of the specific charging protocol and therefore would not alter the relative performance comparison among strategies.

## Author Contributions

M.L. and A.B. conceived the study and developed the methodology. M.L. performed the formal analysis and implemented the model. F.G.Q., D.C., and C.S. carried out electrical tests. M.L., F.B., and M.P. conducted teardown and electrochemical characterization. A.R. and U.N.M. assisted with Monte Carlo code development and related investigation. F.B., M.P., G.L., and M.P.C. provided key resources and supervised experimental activities. A.B. supervised, administered, and funded the project. M.L. and A.B. wrote the original draft of the manuscript, and all co‐authors contributed to its review.

## Conflicts of Interest

The authors declare no conflict of interest.

## Supporting information




**Supporting File**: advs75306‐sup‐0001‐SuppMat.pdf.

## Data Availability

All battery datasets containing raw experimental data are available at the following repository (10.17632/jnnxmt35sy.1). Source data are provided with the paper. The code for the P2D phase‐field electrochemical model is available from the corresponding authors upon reasonable request.
