# Supplementary material for: Faster and Durable: A Cell‐to‐System Validation of a Low‐Degradation Fast‐Charge Protocol for Li‐Ion Batteries
Source: Adv Sci (Weinh). 2026 Apr 17;13(38):e75306. doi: 10.1002/advs.75306 (PMC13335495; doi:10.1002/advs.75306)
Supplement: Supplementary file 1 — Supporting File: advs75306‐sup‐0001‐SuppMat.pdf. [file ADVS-13-e75306-s001.pdf]

**Supplementary Information**

# Faster and durable: a cell-to-system validation of a low-degradation fast-charge protocol for Li-ion batteries

Marco Lagnoni[1,*], Francesco Giuseppe Quilici[2], Davide Cademartori[3], Claudio Scarpelli[2], Uways Nurulain Mithoowani[4], Federica Barontini[1], Alessandro Ruvio[4], Monica Puccini[1], Giovanni Lutzemberger[2], Maria Paola Carpanese[3], Antonio Bertei[1,*]

*[1]Department of Civil and Industrial Engineering, University of Pisa, Pisa, Italy*

*[2]Department of Energy, Systems, Territory and Constructions Engineering, University of Pisa, Pisa, Italy*

*[3]Department of Civil, Chemical and Environmental Engineering, University of Genoa, Genova, Italy*

*[4]Department of Astronautics, Electrical and Energetic Engineering, Sapienza University of Rome, Rome, Italy*

*Corresponding authors

marco.lagnoni@unipi.it

antonio.bertei@unipi.it

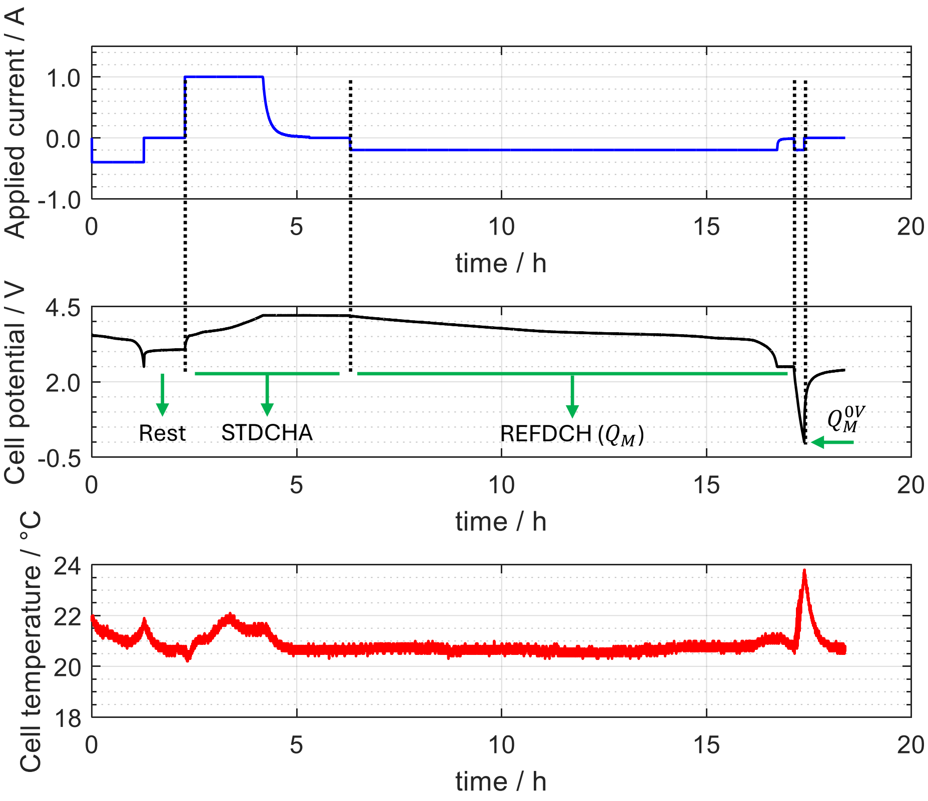


**Figure S1. Current, voltage, and temperature evolution during the experimental procedures: example of the standard charge (STDCHA) and reference discharge (REFDCH) protocols.** The top row shows the applied current profile, the middle row presents the recorded potential response, and the bottom row displays the battery temperature profile. Dashed lines in the current and potential plots indicate the transitions between different experimental phases: rest, STDCHA, and REFDCH. Additionally, a discharge to 0 V is included, as required before teardown analysis for safety reasons and to extract the maximum capacity at 0 V ($Q_M^{0V}$) required to reconstruct the state-of-lithiation at electrodes at 100% and 0% state-of-charge (SoC) and 0 V (refer to Figure S10).

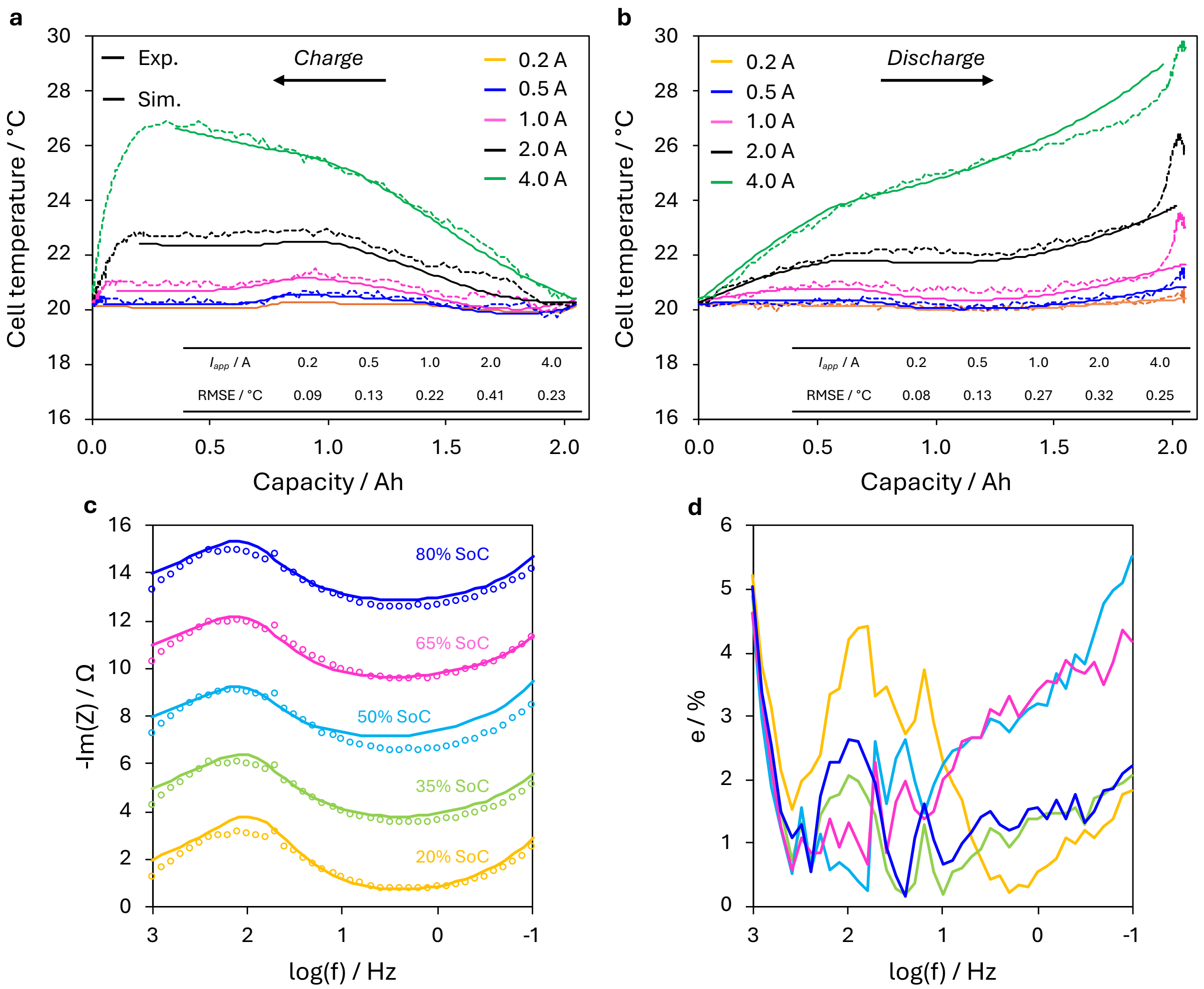


**Figure S2. Comparison between model and experiments for temperature evolution and electrochemical impedance spectroscopy at different SoCs.** (a, b) Comparison of model-predicted (solid lines, constant current phase only) and experimental (dashed lines) battery temperature, with the inset table reporting the root mean square error (RMSE) across 20-80% SoC; arrows indicate the progress of the operation: charge (left) and discharge (right). (c) Comparison of model-predicted (solid lines) and experimental (markers) imaginary part of the battery impedance versus frequency at different SoCs. (d) Frequency-dependent impedance relative error at different SoC levels defined as $e(f) = \left|Z_{sim}(f) - Z_{exp}(f)\right| / \left|Z_{exp}(f)\right|$.

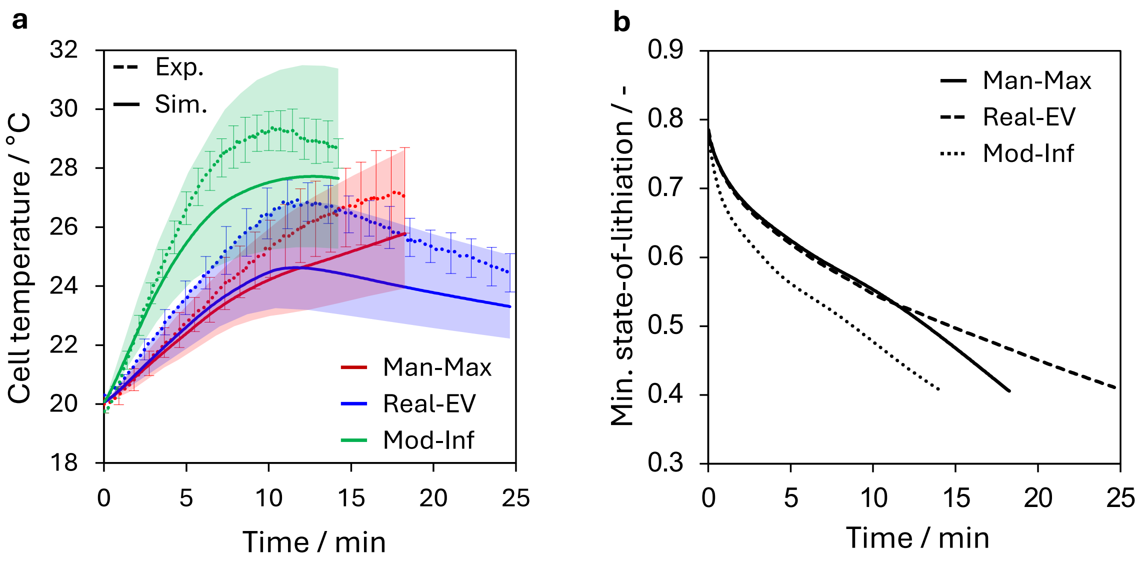


**Figure S3. Model validation and comparison of manufacturer, real EV, and model-informed fast-charge protocols.** (a) Comparison of simulated (solid lines) and experimental (dotted lines) battery temperature evolution for three fast-charge protocols: manufacturer maximum charge profile (Man-Max, red), a commercial fast-charge protocol (Real-EV, blue), and the model-informed fast-charge protocol (Mod-Inf, green). Experimental standard deviation is represented as error bars, obtained from three batteries cycled per protocol, while model variability is shown as shaded regions, estimated using a jackknife resampling of calibration data[1,2]. (b) Assessment of over-delithiation in the positive electrode (NMC532) via modelling. In all fast-charge scenarios, the minimum state-of-lithiation in the positive electrode active material particles remains above the threshold of 0.3 to avoid active material structural change and oxygen evolution[3].

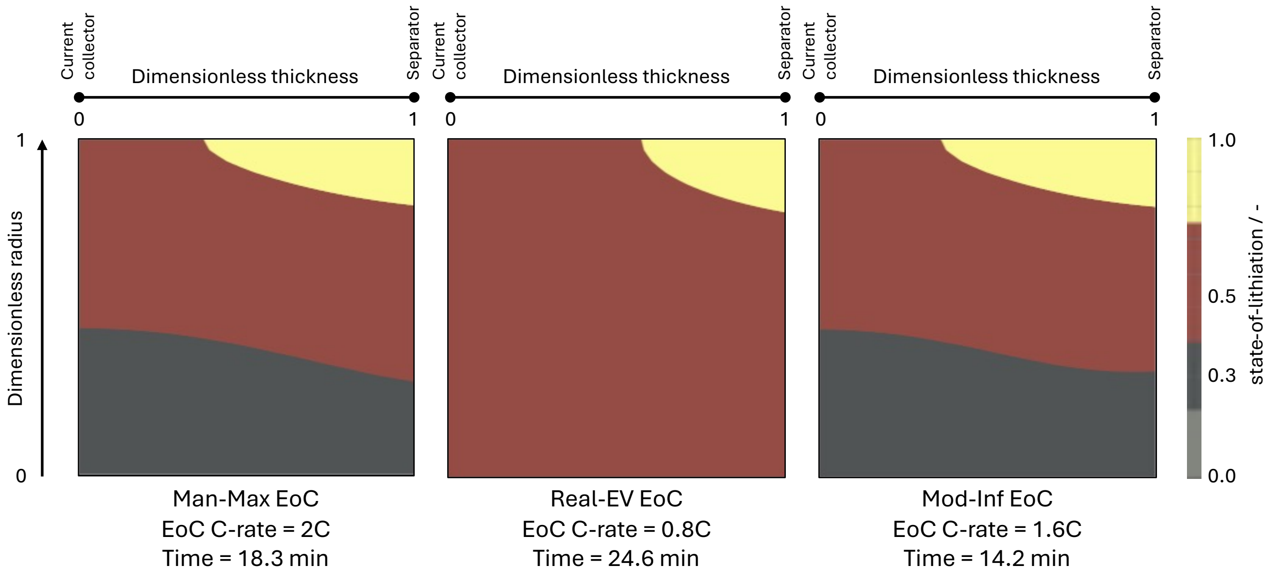


**Figure S4. Phase-field model predictions of staging behaviour in graphite at the end of charge (EoC) under different fast-charge protocols.** The spatial distribution of the state-of-lithiation is shown over a dimensionless 2D cross-section of the graphite electrode for three protocols: (left) the manufacturer maximum charge profile, (centre) a commercial fast-charge protocol, and (right) the model-informed fast-charge protocol. The colour map illustrates the lithiation stages, highlighting spatial heterogeneity. The corresponding total charge time and C-rate at EoC are reported for each case.

| Battery Cycler: Chroma 17020 Battery Pack Tester | |
|---|---|
| Channels | 8 |
| Voltage Range (V) | 0 - 100 |
| Maximum Current (per channel) (A) | 50 |
| Maximum Power (per channel) (kW) | 2.5 |
| Voltage Accuracy | ± 0.02% voltage ± 0.02% voltage range |
| Voltage Resolution (mV) | 3 |
| Current Accuracy | ± 0.05% current ± 0.05% current range |
| Current Resolution (mA) | 2 |
| Temperature Range (°C) | 0 - 90 |
| Temperature Accuracy (°C) | ± 2 |
| Temperature Resolution (°C) | 0.1 |

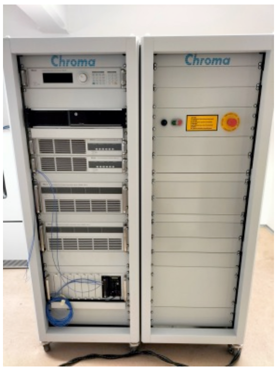


| Climatic Chamber: FDM300 | |
|---|---|
| Manufacturer | FDM |
| Temperature Range (°C) | 0 - 70 |
| Internal Volume (L) | 370 |
| Max set-point temperature oscillation (°C) | ± 0.5 |

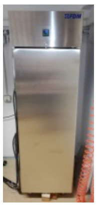

**Figure S5. Experimental setup specifications I.** Specifications of the battery cycler, climatic chamber, and thermocouples used in the DESTEC Battery Lab.

| Potentiostat/Galvanostat: Autolab PGSTAT302N + FRA32M | |
|---|---|
| Max number of modules | 8 |
| Voltage Range [V] | -10 - 10 |
| Maximum Current [A] | ±2 |
| Number of current ranges | 9 |
| Number of current ranges remarks | 10 nA to 1 A |
| Potential and current accuracy | V: ± 0.2% ± 2 mV and i: ± 0.2% ± 0.2% of current range |
| Potential resolution | 0.3 μV (gain 1000) |
| Current resolution | 0.0003% (of current range) |
| Maximum bandwidth in Hz | 1 MHz |
| Frequency range | 10 μHz – 1 MHz |
| Frequency resolution | 0.003% |

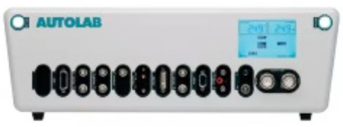


**Figure S6. Experimental setup specifications II.** Specifications of the potentiostat/galvanostat and frequency response analyser used in the DICCA laboratory.

# Battery teardown and characterisation analysis

The teardown analysis systematically characterises the mesoscopic and microscopic properties of the battery, quantifying key parameters essential for accurate electrochemical model parametrisation. This analysis was applied to three commercial 18650 lithium-ion cells (Samsung SDI, nominal capacity 2 Ah), and consists of five main tasks, summarised schematically in Figure S7-10.

The battery disassembly begins by completely discharging cells to 0 V, ensuring safety and enabling determination of the maximum extractable capacity at 0 V ($Q_M^{0V}$). Following discharge, cell dimensions are measured using a caliper, and mass is recorded with a precision balance. Subsequently, upon battery opening in an inert glove box atmosphere, the jelly-roll is extracted, with its volume measured prior to unrolling. This measurement ensures structural integrity and serves as a critical parameter for subsequent compatibility assessments. The jelly-roll, comprising an outer separator (*out*), negative electrode (*N*), internal separator (*S or in*), and positive electrode (*P*), is then unrolled, and each component is separated, washed with dimethyl carbonate (DMC), dimensionally characterised, and prepared for further analyses. Specifically, the geometric area of each electrode is quantified, distinguishing clearly between fully coated (double-coated) and uncoated regions. This procedure was replicated across three cells to quantify inter-cell variability, with intra-cell variability evaluated through repeated measurements at three distinct positions (left, centre, and right) of each sheet, corresponding to the purple markers in the figures below.

To quantitatively characterise electrode composition, a systematic protocol was followed (Figure S8). Electrode samples from three distinct positions (left, centre, and right) were collected, with each sample mass and area measured. Thermogravimetric analysis (TGA) was conducted to measure the mass fractions of the binder and conductive carbon. For the negative electrode, TGA quantified the binder mass fraction ($w_{bd,N}$), whereas for the positive electrode, it separately quantified the binder ($w_{bd,P}$) and conductive carbon ($w_{c,P}$) mass fractions. Following TGA, inductively coupled plasma optical emission spectroscopy (ICP-OES) analysis was performed to determine the elemental composition, revealing the cathode stoichiometry as lithiated $Ni_{0.5}Mn_{0.3}Co_{0.2}O_2$ (NMC532). Combining ICP-OES data with geometric and gravimetric measurements, including TGA-derived fractions, the areal mass loading ($l_i$) of each electrode component was computed.

Thicknesses and volume fractions of electrode components were evaluated using measurements of areal mass loadings and known material densities ($\rho_i$), according to the following relations (Figure S9):

$$L_j = \frac{\sum_i^n \frac{l_{i,j}}{\rho_{i,j}}}{(1-\varepsilon_{ey,j})} \quad (1)$$

$$\varepsilon_{i,j} = \frac{l_{i,j}}{\rho_{i,j} L_j} \quad (2)$$

Here, $L_j$ represents the electrode thickness (*j* = *N, P*), and $\varepsilon_{i,j}$ indicates the volume fractions of graphite (*gr*), NMC532 oxide (*NMCO₂*, Li excluded), binder (*bd*), and conductive carbon (*c*). The only unknown parameter, pore phase volume fraction ($\varepsilon_{ey,j}$), was derived by imposing two constraints: compatibility with total jelly-roll volume and the summation of all electrode phase fractions. These calculations integrated experimentally verified separator porosity and collector thicknesses (provided by manufacturer datasheets). Electrode porosity was determined as approximately 0.28, consistent with literature data[4–6].

State-of-lithiation ($\tilde{c}_s$) quantification for each electrode was performed by combining ICP-OES analysis results and electrochemical discharge data (Figure S9). ICP-OES provided direct measurements of the moles of the host active materials ($n_{gr}$: unlithiated graphite in the negative

electrode; $n_{NMCO_2}$: unlithiated NMC532 oxide in the positive electrode), as well as the corresponding lithium moles ($n_{Li}$) at both electrodes at the fully discharged state of 0 V. At this potential, lithium in the positive electrode ($n_{Li,P}^{0V}$) corresponds to the maximum accessible lithium content, whereas lithium detected in the negative electrode ($n_{Li,N}^{0V}$) is attributed entirely to irreversible losses associated with the solid electrolyte interphase (SEI) formation, leading to an initial state-of-lithiation defined as $\tilde{c}_{s,N}^{0V} = 0$. Our analysis indicates that the amount of lithium lost to SEI formation is approximately equivalent to 0.2 Ah, accounting for ca. 9% of the initial capacity loss. This value aligns with typical first-cycle losses observed in graphitic electrodes[4].

To determine electrode states-of-lithiation at a specific state-of-charge (SoC), electrochemical measurements of accessible capacities ($Q_M$, corresponding to 0% SoC at cell voltage of 2.5 V after REFDCH, and $Q_M^{0V}$, the maximum capacity extracted at 0 V, see Figure S1) were combined with ICP-OES mole measurements. The negative electrode states-of-lithiation at 0% and 100% SoCs were calculated explicitly as follows:

$$\tilde{c}_{s,N}^{min} = \frac{(Q_M^{0V} - Q_M)}{n_{gr}F} \quad (3)$$

$$\tilde{c}_{s,N}^{max} = \tilde{c}_{s,N}^{min} + \frac{Q_M}{n_{gr}F} \quad (4)$$

where $F$ is the Faraday constant. Similarly, the positive electrode state-of-lithiation at 0% and 100% SoC was calculated by combining the ICP-OES data with electrochemical capacities, according to:

$$\tilde{c}_{s,P}^{max} = \tilde{c}_{s,P}^{0V} - \frac{Q_M^{0V} - Q_M}{n_{NMCO_2}F} \quad (5)$$

$$\tilde{c}_{s,P}^{min} = \tilde{c}_{s,P}^{max} - \frac{Q_M}{n_{NMCO_2}F} \quad (6)$$

Finally, Figure S11 presents the particle size distribution (PSD) analysis conducted using SEM imaging coupled with image-based quantification. SEM images (Figure S11a, b) show graphite (negative electrode) and NMC532 (positive electrode) morphologies, while image-analysis (Figure S11c, d) provides PSDs across three cells to ensure statistical reliability, with results reported in terms of the median diameter $d_{50}$ (volume basis) and standard deviation of secondary particles. The impact of segmentation methods was also assessed: automated and human-aided segmentation methods agreed closely for the positive electrode, whereas automatic segmentation underestimated negative electrode particle size due to particle overlap. Human-aided segmentation improved particle size accuracy for the negative electrode, with results remaining within the variance observed from automatic analysis of all cells (Figure S11e).

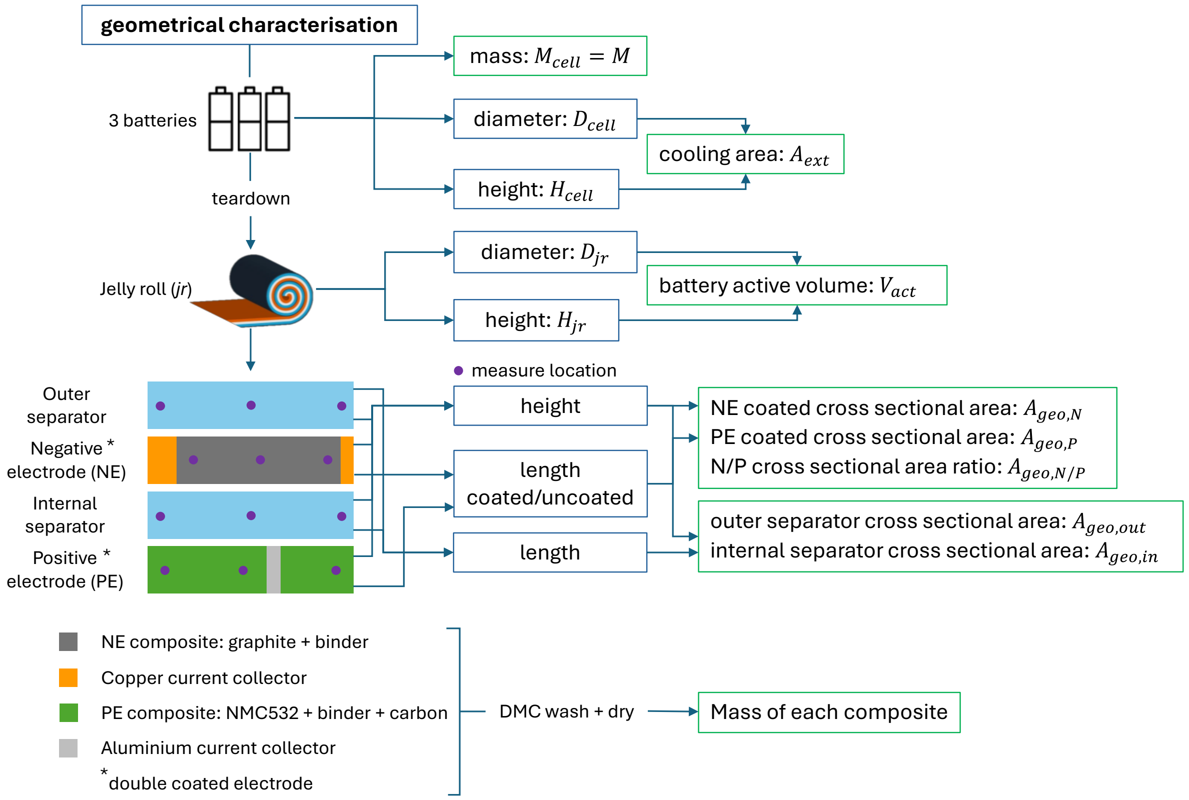


**Figure S7. Workflow schematics for battery macroscopic geometrical characterisation.** The schematics outlines the approach used to perform the teardown analysis of the batteries and extract key macroscopic parameters.

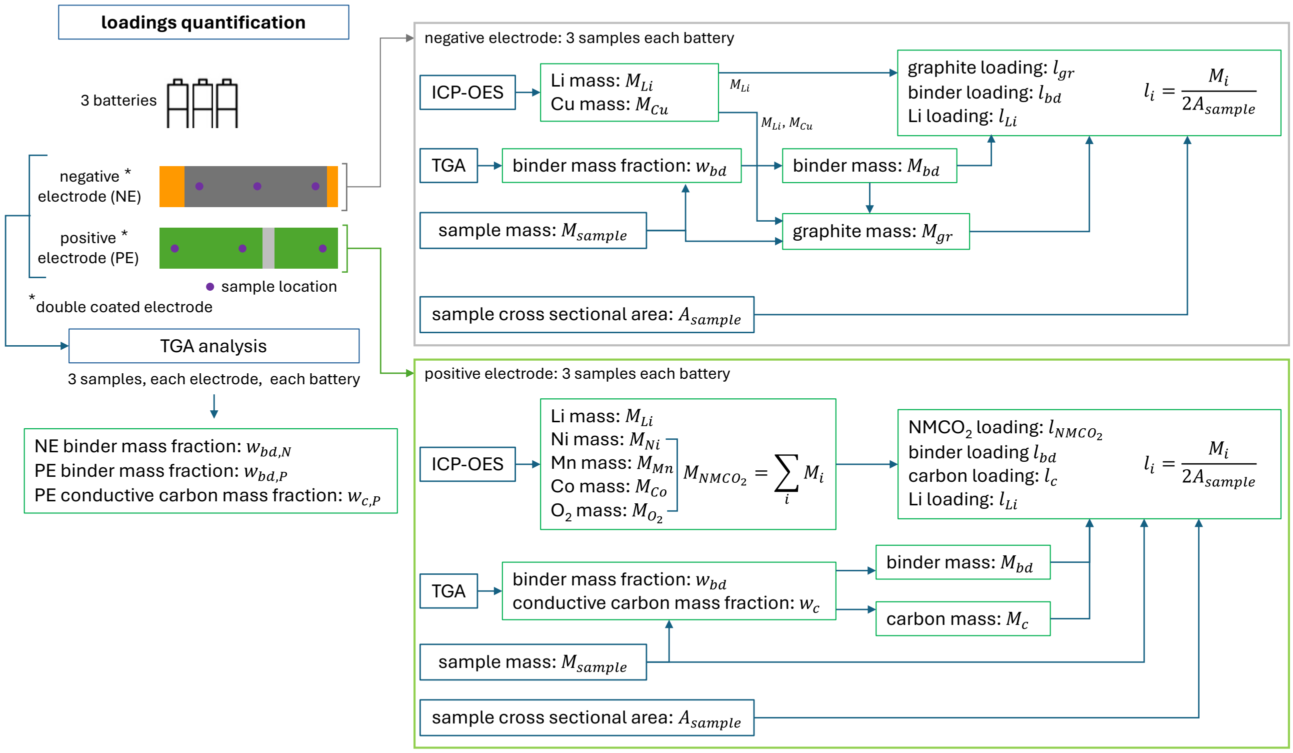


**Figure S8. Workflow schematics for battery loading characterisation at electrodes.** The schematics outlines the approach used to measure the single components loading of the negative and positive electrodes. The process involved the utilisation of different techniques such as inductively coupled plasma optical emission spectrometry (ICP-OES) and thermogravimetric analysis (TGA).

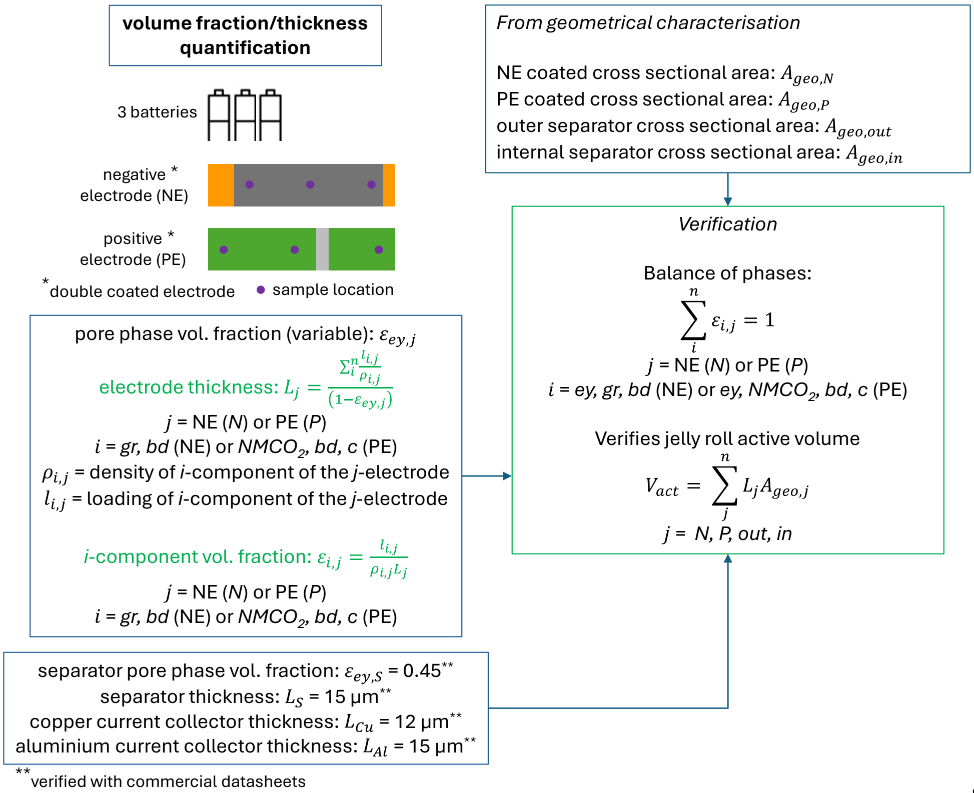


**Figure S9. Workflow schematics for electrode volume fractions and thickness quantification.** The schematic outlines the steps and data required for quantifying the volume fraction of phases in electrodes and the electrode thicknesses as well. This procedure relies on data obtained from previous analyses. Verification of the consistency of the calculated parameters was performed by assessing the internal volume of the jelly-roll and ensuring phase balance within the electrodes.

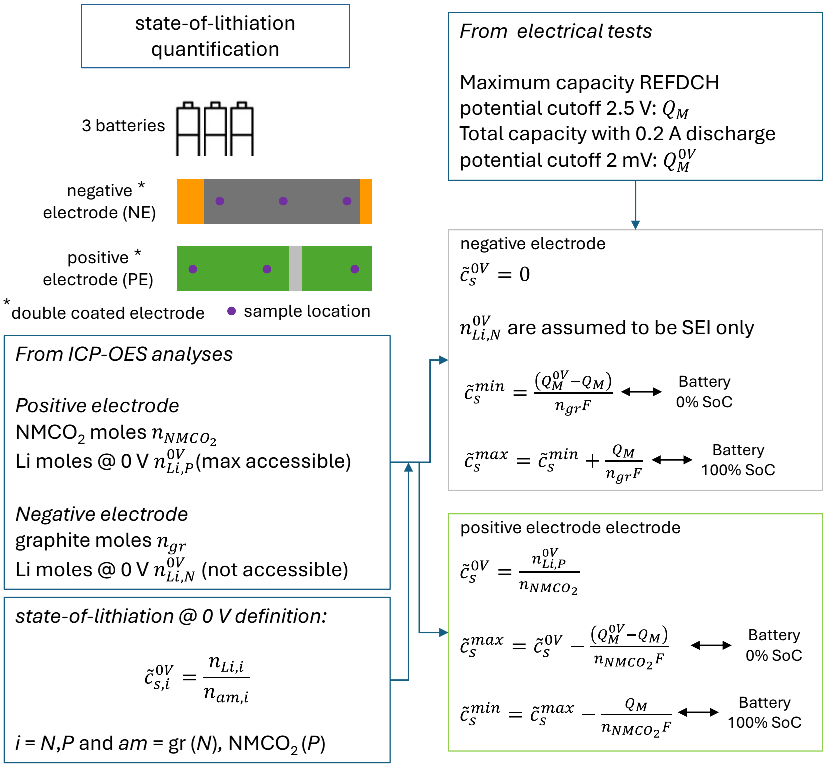


**Figure S10. Workflow schematics for state-of-lithiation quantification at electrodes.** The schematic outlines the sequence of calculations used to quantify the state-of-lithiation at the electrodes, based on a combination of characterisation techniques including ICP-OES analysis and electrical data. The determined states-of-lithiation fully characterise the lithium content in the active material at 0 V, 0% and 100% SoC.

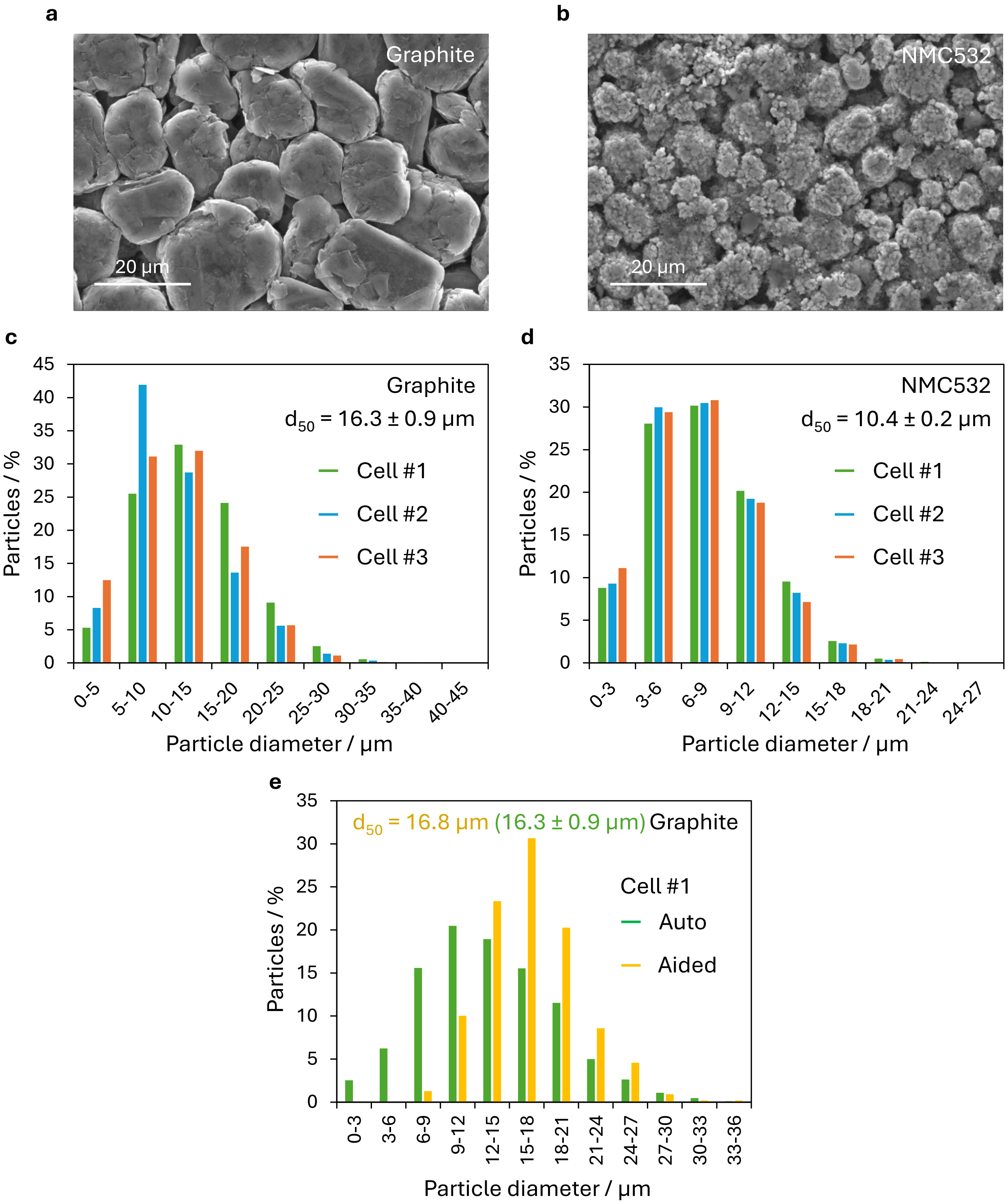


**Figure S11. Particle size distribution analysis of electrode active materials.** (a, b) Scanning electron microscopy (SEM) images of the negative and positive electrode composites, featuring graphite and NMC532 active materials, respectively. (c, d) Particle size distributions obtained from image analysis using ImageJ software on 2D top-view SEM images. Particle size analysis was performed on SEM images from three different cells to ensure statistical reliability, with results reported as volume-averaged median ($d_{50}$) and standard deviation. (e) Comparison of the particle size distribution for the graphitic electrode using automatic (green) and human-aided (yellow) segmentation approaches. Human-aided segmentation was performed only for cell #1 and not repeated for cells #2 and #3, as the results fell within the measurement uncertainty. In the case of the graphite electrode, automatic segmentation led to an underestimation of the median particle size due to particle overlap, whereas for the NMC electrode, the results from automatic and human-aided segmentation agreed.

## Mathematical framework

The following figure and tables summarise schematics, key equations, and boundary/initial conditions of the model used in this work and consolidated in other studies conducted by our group[4,7–9]. The model stems from the Pseudo-2-Dimensional (P2D) model introduced by Newman et al.[10] which is based on porous electrode theory. Such approach offers a homogenised mesoscopic description of charge and mass conservation in both the solid ($s$, active material) and liquid ($ey$, electrolyte) phases[11]. By representing the battery unit cell as a continuum, it incorporates microstructural details (such as volume fraction $\varepsilon$, tortuosity factor $\tau$, particle radius $r_p$, and specific active material surface area $A_{am}$) via effective transport and kinetic parameters.

The unit cell modelled in this work is shown in Figure S12, comprising current collectors (*CC*), negative (*N*) and positive (*P*) electrodes, and a separator (*S*), each with a specific thickness ($L_i$). Note that electron transport in the current collectors is neglected in this study and is therefore omitted from the model domains. For the battery under investigation, the negative electrode contains graphite, where the solid phases in the electrode composite are graphite (*s*) and binder (*bd*), with graphite serving as the electron-conducting medium (*ed*). The positive electrode contains NMC532, where the solid phases include NMC532 as the active material (*s*), binder (*bd*), and conductive carbon (*c*) as the electron-conducting phase (*ed*). The model couples a 1D spatial coordinate *x* along the through-thickness direction with a pseudo-1D radial coordinate *y* to capture the solid-state Li transport within active material particles.

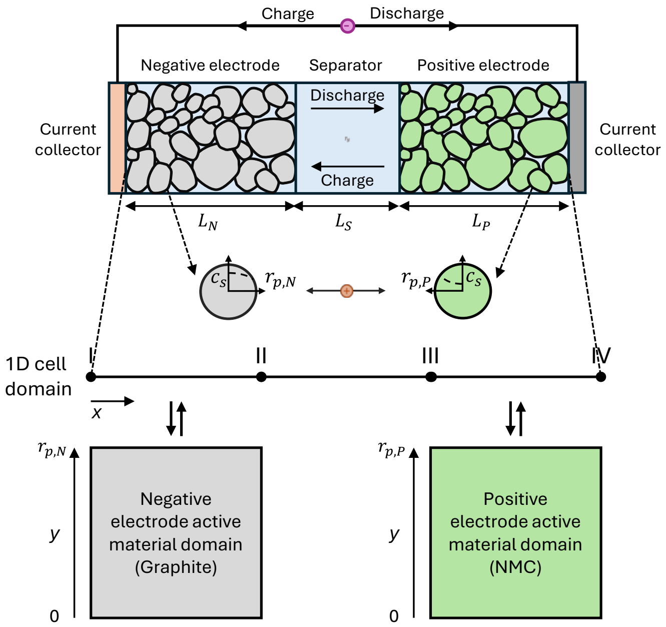


**Figure S12. Battery unit cell and model schematics.** Representation of the battery unit cell and the mathematical domains of the model.

Along the *x* direction, the model computes ion transport in the electrolyte using concentrated solution theory[12,13]. This implies solving for the reduced electrochemical potential ($\tilde{\mu}_+^*$) and concentration ($c$) of Li-ions via microscopic balance equations for mass (equation (7), written as charge balance with one electron reaction) and charge (equation (9)) in the liquid phase, provided the definition of the Li-ions diffusional flux ($J_{conc}$, equation (8), charge basis) and the total ionic current in the electrolyte phase ($J_2$, equation (10)). The charge-transfer reaction of Li (de)intercalation at the particle surface couples the through-thickness and radial 1D domains. Such charge-transfer reaction represents a current density at the active material particle surface ($J_{int}$), which is modelled via a Butler-

Volmer kinetics[14] (equations (11)-(13)). In each electrode, the reduced electrochemical potential of electrons ($\tilde{\mu}_e^*$) in the electroconductive phase is solved via Ohm law (equations (16) and (17))

Li solid-state diffusion in both graphite and NMC materials is modelled using a phase-field approach based on non-equilibrium thermodynamics[9,15–18]. While originally introduced to model phase-separating materials[15,16], this approach can also be used to model Li transport in solid-solution materials[4,9,19]. The concentration of intercalated Li in the active material ($c_{s,i}$, with $i = N$ or $P$) is solved with a mass balance as in equation (18), where the divergence operator is explicitly expressed in spherical coordinates. The molar flux of intercalated Li across the particle radius ($N_{s,i}$) follows the definition as in equation (19), where $D_s$ is the solid-state diffusion coefficient, $\tilde{c}_{s,i} = c_{s,i}/c_{s,i}^{max}$ is the state-of-lithiation with $c_{s,i}^{max}$ as the maximum molar concentration at full lithiation of the active material, and $\tilde{\mu}_i$ (equation (20)) is the dimensionless form of intercalated Li chemical potential difference between filled and empty sites[9]. Following this approach $\tilde{\mu}_i$ can be written as the summation of two terms, where $\mu_{td,i}/R_g T$ is the dimensionless chemical potential thermodynamic contribution (i.e., function only of the local concentration of intercalated Li), while $-a_i^2\left(\frac{\partial^2 \tilde{c}_{s,i}}{\partial y^2} + \frac{2}{y}\frac{\partial \tilde{c}_{s,i}}{\partial y}\right)$, which is present only in phase-separating materials (i.e., graphite), represents the non-local contribution to the dimensionless chemical potential, which accounts for the free energy of phase boundaries according to a Cahn-Hilliard phase-field framework[15,20–22]. It is worth noting that the quantity $a_i$ indicates the characteristic length of the phase boundary between Li-rich and Li-poor phases. This framework allows the usage of the same functional form of the solid-state molar flux for both phase-separating (graphite) and solid-solution (NMC) materials[9], which in turn allows for direct comparison of critical properties such as the solid-state diffusion coefficient (Figure S15). The dimensionless chemical potential $\tilde{\mu}_i$ not only determine Li solid-state diffusion but also the rate of charge-transfer kinetics of lithium intercalation. In fact, at each electrode, the electric equilibrium potential ($E_{eq,i}$) follows the Nernst law as in equation (13), where $E_i^\Theta$ is the standard electric potential of intercalation in the active material. At particle surface, the calculation of the equilibrium potential contributes to the activation overpotential of the charge-transfer reaction ($\eta_{int,i}$) as shown in equation (12). Additionally, for EIS analysis, a double-layer capacitance ($C_{dl,i}$) is coupled to the intercalation process (equation (14)). The reduced electrochemical potential difference at electrode/electrolyte interface ($\Delta E_N = \Delta\tilde{\mu}_{e,+}^*$) is also introduced at the negative electrode only (equation (15)) as an indicator of the battery degradation propensity since the rate of both Li plating and solid-electrolyte interface growth depend explicitly on it[7,23–26].

Temperature change in the battery is considered by solving a lumped energy conservation equation, as the temperature distribution within the unit cell can be approximated as uniform[27]. The cell thermal evolution is simulated via a single temperature value $T$, which depicts the average temperature of the battery over time, and a unique cell thermal mass ($Mc_p$) using equations (21) and (22). In equation (21), the quantity $hA_{ext}(T - T_{amb})$ represents the heat removed by convection, while $\dot{q}_{gen}$ is the total volumetric heat generated in the unit cell. The latter is given by the summation of the volumetric heat generated in the negative and positive electrodes, and in the separator, as in equation (22). Notably, the current collectors volumetric heat generation has been neglected in this study as its contribution, that is purely ohmic, is small compared to that of the other unit cell components given the very high electrical conductivity of the current collector materials[6,28,29]. Each volumetric contribution from the unit cell components is obtained by integral averaging the specific heat per unit of cross-sectional area generated in each domain over the corresponding thickness (equation (23)). Following the well-established framework introduced by Bernardi et al.[30], three major heat contributions define the heat generated in the unit cell: i. the reversible heat $\dot{Q}_{rev}$ (equation (24)) associated to the entropic change of charge-transfer phenomena, ii. the irreversible heat $\dot{Q}_{rxn}$ (equation (25)) associated to the activation overpotential of electrochemical reactions, and iii. the ohmic heat $\dot{Q}_{ohm}$ (equation (26)) associated to

the Joule dissipations occurring in electrolyte and solid phases. By solving the thermal model, the temperature evolution of the battery can be coupled to the electrochemical model to update equilibrium, transport, and kinetics parameters over time during the simulation (equations (27)-(29)).

The P2D thermo-electrochemical model is well consolidated in the literature. The reader is referred to specific papers and textbooks for their derivation[6,9–11,13,30], only a brief description of the model key equations and quantities is reported in Table S1; detailed boundary conditions for the electrochemical model and initial conditions are outlined in Table S2 and Table S3, respectively. Corresponding nomenclature is provided at the end of the supplementary information.

**Table S1.** Governing equations and key quantities of the phase-field P2D electrochemical model and 0D lumped thermal model (continues).

| **1D+1D electrochemical model framework** | |
|---|---|
| *Electrolyte phase, i = N, S, P* | |
| $\varepsilon_{ey,i} F \frac{\partial c}{\partial t} + \frac{\partial}{\partial x}(J_{conc}) = (1-t_+) J_{int,i} A_{am,i}$ | (7) |
| $J_{conc} = -\frac{\varepsilon_{ey,i}}{\tau_{ey,i}} F \tilde{D} \frac{\partial c}{\partial x}$ | (8) |
| $\frac{\partial}{\partial x}(J_2) = J_{int,i} A_{am,i}$ | (9) |
| $J_2 = -\frac{\varepsilon_{ey,i}}{\tau_{ey,i}} \sigma \frac{\partial \tilde{\mu}_+^*}{\partial x} + \frac{\varepsilon_{ey,i}}{\tau_{ey,i}} \sigma \frac{2R_gT}{F}(1-t_+)\gamma_\pm \frac{\partial lnc}{\partial x}$ | (10) |
| *Interfacial equations, i = N, P* | |
| $J_{int,i} = k_{int,i}\left(\frac{c}{c^\theta}\right)^\alpha \left(1-\tilde{c}_{s,i}\right)^\alpha \tilde{c}_{s,i}{}^{(1-\alpha)}\left[exp\left(\frac{\alpha F}{R_gT}\eta_{int,i}\right) - exp\left(-\frac{(1-\alpha)F}{R_gT}\eta_{int,i}\right)\right]$ | (11) |
| $\eta_{int,i} = \tilde{\mu}_{e,i}^* - \tilde{\mu}_+^* - E_{eq,i}$ | (12) |
| $E_{eq,i} = E_i^\theta - \frac{R_gT}{F}\tilde{\mu}_i$ | (13) |
| $J_{dl,i} = C_{dl,i}\left(\tilde{\mu}_{e,i}^* - \tilde{\mu}_+^*\right)$ | (14) |
| $\Delta E_i = \tilde{\mu}_{e,i}^* - \tilde{\mu}_+^*$ | (15) |
| *Electroconductive phase, i = N, P* | |
| $\frac{\partial}{\partial x}\left(J_{1,i}\right) = -J_{int,i} A_{am,i}$ | (16) |
| $J_{1,i} = -\frac{\varepsilon_{ed,i}}{\tau_{ed,i}}\sigma_{e,i}\frac{\partial \tilde{\mu}_{e,i}^*}{\partial x} = -\sigma_{e,eff,i}\frac{\partial \tilde{\mu}_{e,i}^*}{\partial x}$ | (17) |
| *Active material phase, i = N, P* | |
| $\frac{\partial c_{s,i}}{\partial t} + \frac{\partial}{\partial y}\left(N_{s,i}\right) = -\frac{2}{y}N_{s,i}$ | (18) |
| $N_{s,i} = -D_{s,i} c_{s,i}^{max} \tilde{c}_{s,i}\left(1-\tilde{c}_{s,i}\right)\frac{\partial \tilde{\mu}_i}{\partial y}$ | (19) |
| $\tilde{\mu}_i = \frac{\mu_i}{R_gT} = \frac{\mu_{td,i}}{R_gT} - a_i^2\left(\frac{\partial^2 \tilde{c}_{s,i}}{\partial y^2} + \frac{2}{y}\frac{\partial \tilde{c}_{s,i}}{\partial y}\right)$ *i = N* and $\tilde{\mu}_i = \frac{\mu_i}{R_gT} = \frac{\mu_{td,i}}{R_gT}$ *i = P* | (20) |

| | |
|---|---|
| **0D lumped thermal model framework** | |
| $Mc_p \frac{dT}{dt} = \dot{q}_{gen} L_{tot} A_{act} - hA_{ext}(T - T_{amb})$ | (21) |
| $\dot{q}_{gen} = (\dot{q}_N + \dot{q}_S + \dot{q}_P)$ | (22) |
| $\dot{q}_i = \int(\dot{Q}_{rev,i} + \dot{Q}_{rxn,i} + \dot{Q}_{ohm,i}) \frac{dx}{L_{tot}}$ *i* = *N*, *S*, *P* and $L_{tot} = L_N + L_S + L_P$ | (23) |
| $\dot{Q}_{rev,i} = J_{int,i} A_{am,i} T EHC_i$ *i* = *N*, *P* | (24) |
| $\dot{Q}_{rxn,i} = J_{int,i} A_{am,i} \eta_{int,i}$ *i* = *N*, *P* | (25) |
| $\dot{Q}_{ohm,i} = \frac{\varepsilon_{el,i}}{\tau_{el,i}} \sigma_{el,i} \left(\frac{\partial \tilde{\mu}^*_{e,i}}{\partial x}\right)^2 + \frac{\varepsilon_{ey,i}}{\tau_{ey,i}} \sigma \left(\frac{\partial \tilde{\mu}^*_{+}}{\partial x}\right)^2 - \frac{\varepsilon_{ey,i}}{\tau_{ey,i}} \frac{2RT\sigma}{F} (1 - t_+) \gamma_\pm \frac{\partial lnc}{\partial x} \frac{\partial \tilde{\mu}^*_{+}}{\partial x}$ *i* = *N*, *S*, *P* | (26) |
| **Couplings for equilibrium, transport, and kinetics parameters with temperature change** | |
| $E_i^\theta = E_i^{\theta,\circ} + (T - T^\circ) EHC_i$ *i* = *N*, *P* | (27) |
| $D_{s,i} = D_{s,i}^\circ exp\left(-\frac{E_{D_{s,i}}}{R_g}\left(\frac{1}{T} - \frac{1}{T^\circ}\right)\right)$ *i* = *N*, *P* | (28) |
| $k_{int,i} = k_{int,i}^\circ exp\left(-\frac{E_{k_{int,i}}}{R_g}\left(\frac{1}{T} - \frac{1}{T^\circ}\right)\right)$ *i* = *N*, *P* | (29) |

**Table S2.** Boundary conditions of the phase-field P2D electrochemical model.

| | |
|---|---|
| Balance of mass – *ey* phase (equation (7)) | $J_{conc}\vert_{x=I} = 0;\ J_{conc}\vert_{x=IV} = 0$ |
| Balance of charge – *ey* phase (equation (9)) | $J_2\vert_{x=I} = 0;\ J_2\vert_{x=IV} = 0$ |
| Balance of charge – *el* phase (equation (16)) | $\tilde{\mu}^*_{e,N}\vert_{x=I} = 0;\ J_{1,N}\vert_{x=II} = 0$<br>$J_{1,P}\vert_{x=IV} = \frac{i_{app}}{A_{act}}\ (CC)$<br>$J_{1,P}\vert_{x=III} = 0;\ \tilde{\mu}^*_{e,P}\vert_{x=IV} = V_{CV}\ (CV)$<br>$J_{1,P}\vert_{x=IV} = \frac{i_{pert}}{A_{act}}\ (EIS)$ |
| Balance of mass – *s* phase (equation (18)) | $J_{s,i}\vert_{y=0} = 0;\ FN_{s,i}\vert_{y=r_{p,i}} = J_{int,i}$ |

**Table S3.** Initial conditions of the phase-field P2D electrochemical model 0D lumped thermal model dependent variables.

| | |
|---|---|
| $c$ – $Li^+$ molar concentration | $c = c^{in}$ |
| $\tilde{\mu}^*_+$ – $Li^+$ reduced electrochemical potential | $\tilde{\mu}^*_+ = -E_{eq,N}$ |
| $\tilde{\mu}^*_{e,i}$ – $e^-$ reduced electrochemical potential | $\tilde{\mu}^*_{e,N} = 0\ V$<br>$\tilde{\mu}^*_{e,P} = E_{eq,P} - E_{eq,N}$ |
| $c_{s,i}$ – Intercalated Li molar concentration | $c_{s,N} = \tilde{c}^i_{s,N} c^{max}_{s,N}$ with *i* = min (if charge) or max (if discharge)<br>$c_{s,P} = \tilde{c}^i_{s,P} c^{max}_{s,P}$ with *i* = max (if charge) or min (if discharge) |
| $T$ – Battery temperature | $T = T_{amb}$ |

# Model parametrisation

The accuracy and reliability of the described mathematical framework are intrinsically dependent upon the precise quantification of its governing parameters. Therefore, a systematic approach was adopted to identify, measure, and calibrate all necessary model parameters, establishing a direct connection between the theoretical framework and experimental data obtained through detailed experimental teardown and characterisation. The model parameters are categorised into two groups: those experimentally determined (e.g., geometrical dimensions, electrode composition, and thicknesses) and those not directly measurable, which require estimation through model fitting, including solid-phase diffusion coefficients and charge-transfer kinetics. During experimental characterisation, median values and uncertainty ranges were established for all accessible parameters, providing a solid foundation for model calibration. This rigorous methodology enabled fine-tuning of model parameters within experimentally validated ranges. For unknown parameters, dedicated experiments such as electrochemical impedance spectroscopy (EIS), combined with statistical approaches (e.g., Jackknife resampling), were employed to quantify uncertainties. This comprehensive parameterisation strategy ensured that the final model maintained physical consistency and predictive reliability.

Calibration of experimentally measured parameters began using their median values as initial guesses. Particle radii ($r_p$) were set to the median particle size distribution (PSD) values obtained from SEM analyses (Figure S11). The external surface area ($A_{ext}$), defining the thermal exchange interface with the climatic chamber environment, and total cell mass ($M$) were directly obtained from measurements illustrated in Figure S7. Mesoscopic characterisation (Figure S7) showed that geometric cross-sectional areas of negative ($A_{geo,N}$) and positive electrodes ($A_{geo,P}$) differed, with the negative electrode presenting a larger geometric area. In the model, the cross-sectional active area was assumed equal to the positive electrode geometric area ($A_{geo,P} = A_{act}$) for both electrodes. Consequently, experimentally measured loading values for the negative electrode were updated by multiplying them by the geometric area ratio ($A_{act,N/P}$), ensuring all active graphite material was accurately represented. For the positive electrode, experimentally obtained loading values (Figure S8) were adjusted based on the difference between capacities extracted via the REFDCH protocol from cells tested with calibration cycles and cells discharged prior to teardown. Starting from these updated loadings and following the procedure described in Figure S9, electrode thicknesses ($L$) and phase volume fractions ($\varepsilon$) were computed, with pore-phase volume fraction ($\varepsilon_{ey}$) serving as input. Additionally, the specific active surface area per unit electrode volume ($A_{am}$) was calculated as $3\varepsilon_{am}/r_p$. Effective transport properties were derived using Bruggeman approximation, specifying Bruggeman coefficients ($\beta$) for electronic conductive phases (graphite in negative electrode, conductive carbon and NMC532 in positive electrode) and ionic conductive phase (pore phase).

States-of-lithiation at 0 V, 0% SoC, and 100% SoC were calculated using the methodology outlined in Figure S10, with specific capacity values $Q_M$ and $Q_M^{0V}$ reported in Table S4. Minor adjustments to $Q_M$ and $Q_M^{0V}$ were made during cell open circuit potential (OCP) calibration to ensure simulation results closely matched experimental OCP measurements (Figure S13). Considering transport properties, the charge-transfer kinetic constant ($\mathring{k}_{int,N}$) for lithium intercalation at the negative electrode initially referenced literature values (Ahn et al.[4]) and is subsequently calibrated to fit model predictions to experimental charge/discharge and EIS data (Manuscript Figure 1). The lithium diffusion coefficient in graphite ($\mathring{D}_{s,N}$) was taken from Lu et al.[7] and adapted to fit impedance data (EIS) measured at different SoCs (Manuscript Figure 1). Effective electronic conductivity of graphite ($\sigma_{ed,eff,N}$) was calculated equal to 36 S/m by setting $\beta_{el} = 1.5$. At the positive electrode, both the kinetic constant ($\mathring{k}_{int,P}$) and lithium diffusion coefficient ($\mathring{D}_{s,P}$) were derived from Nguyen et al.[5] and adjusted to fit model

predictions to experimental charge/discharge and EIS data. Effective electronic conductivity for the positive electrode ($\sigma_{ed,eff,P}$) was calculated from the weighted average based on volume fraction of active material and conductive carbon phases and set at 0.79 S/m, also using $\beta_{el} = 1.5$. For both electrodes, Figure S15 reports a direct comparison of the solid-state diffusion coefficients used in this study. The double-layer capacitances for both electrodes ($C_{dl}$) were calibrated using EIS data by fitting imaginary impedance plots (Supplementary Figure S2c), resulting in values of approximately 0.8 F $m^{-2}$ for graphite and 0.2 F $m^{-2}$ for NMC532. To accurately reproduce experimental charge/discharge cycles and EIS data, a constant contact resistance ($R_{el,ext}$) was introduced into the model and calibrated accordingly. The symmetry coefficient ($\alpha$) in Butler-Volmer kinetics was set to 0.5 for both electrodes. Electrolyte transport properties were assumed equal to those reported by Landesfeind et al.[31] for a 1M $LiPF_6$ solution in EC-EMC (3:7 vol%) with 2% FEC, with an initial $Li^+$ concentration ($c^{in}$) set at 1000 mol $m^{-3}$. Jackknife resampling was[32] applied to electrochemical charge/discharge data to determine uncertainty ranges for key parameters, including charge-transfer kinetic constants and the pore-phase Bruggeman coefficients, allowing for the generation of confidence intervals in model predictions (Manuscript Figs. 2 and 3, and Supplementary Figure S3). Notably, the resulting uncertainty bands also account for plausible variations in Bruggeman coefficients for both electrodes (± 0.2), including cases where the graphite negative electrode exhibits higher tortuosity than the NMC positive electrode[32].

Activation energies for kinetic constants ($E_{k_{int}}$) and diffusion coefficients ($E_{D_s}$) were taken from previous reviews by our group, aligning with literature values[6]. Entropic heat coefficients ($EHC$) for temperature dependence of electrode OCPs were directly taken from specific studies, specifically Yazami et al.[33] for graphite and Williford et al.[34] for NMC, and reported in Figure S14. Maximum lithium concentrations in active materials ($c_s^{max}$) were fixed at 31360 mol $m^{-3}$ for graphite and 48989 mol $m^{-3}$ for NMC532, consistent with literature data[6]. Following a phase-field approach, the characteristic interface length ($a$) for graphite was set to approximately 3.16 μm, as in Lu et al.[7], whereas no interfacial length was required for NMC532. Standard electric potentials ($E^{\theta}$) were obtained from fitting experimental OCP data for graphite (Billaud et al.[35]) and by integrating the OCP function provided by Zhu et al.[36] for NMC532 (3.99 V). The heat transfer coefficient ($h$) was calibrated to experimental temperature data from cycling tests and set to 10 W $m^{-2}$ $K^{-1}$. Equivalent specific heat capacity ($c_p$) was calculated from thicknesses, geometric areas, and physical properties of individual battery components following the methodology described by Lagnoni et al.[6], resulting in 945 J $kg^{-1}$ $K^{-1}$, consistent with literature data for graphite-NMC battery cells[37].

Finally, compatibility of all calibrated model parameters with experimental uncertainty ranges obtained from teardown and characterisation was systematically verified. Each parameter successfully fell within its experimentally determined uncertainty interval, providing robust cross-validation between the experimental characterisation and model parametrisation procedures. A list of model parameters is provided in Table S4, with uncertainty intervals evaluated via jackknife resampling for pore phase Bruggeman coefficients and intercalation kinetic constants; values within round brackets refer to averages and standard deviations obtained experimentally via teardown and characterisation analysis.

To ensure full transparency, all model parameters are explicitly classified according to their origin:

- *Measured in this work* (superscripts a–c): directly obtained from teardown, ICP-OES, TGA, SEM, or geometrical measurements (e.g., electrode loadings, porosity, particle sizes, state-of-lithiation).
- *Taken directly from the literature* (superscript d): adopted from consolidated references without further modification (e.g., electrolyte transport properties, maximum lithium concentrations).

- *Adapted from the literature and refined by fitting to this work* (superscript e): initialised from the literature or previous group data and subsequently calibrated against galvanostatic cycling and/or EIS (e.g., kinetic constants, solid-state diffusion coefficients, double-layer capacitances, Bruggeman factors, contact resistance).
- *Uncertainty estimates* (superscript f): obtained by applying jackknife resampling to our experimental calibration data.

Figure S13-15 illustrate literature data together with the fitted curves obtained in this study, and Figure S16 reports the concentration-dependent electrolyte properties taken from Landesfeind et al.[38]. This scheme allows the reader to trace the origin and role of each parameter reported in Table S4.

**Table S4.** Geometrical, microstructural, electrochemical and transport parameters of the phase-field P2D electrochemical model.

| | **Negative electrode** *(graphite)* | **Separator** | **Positive electrode** *(NMC532)* |
|---|---|---|---|
| Thickness<br>$L$ / [μm] | 51.13 [b] | 15.00 [b,h] | 44.20 [b] |
| Particle radius<br>$r_p$ / [μm] | 8.4 [c]<br>($d_{50}$ – from Figure S11e) | - | 5.2 [c]<br>($d_{50}$ – from Figure S11d) |
| Active material surface area per unit of volume<br>$A_{am}$ / [m$^{-1}$] | 2.403·10$^5$ [b] | - | 3.582·10$^5$ [b] |
| Volume fraction<br>$\varepsilon$ / [-] | 0.673 – active material [b]<br>0.039 – binder [b]<br>0.288 – pore [b,g] | 0.450 [5] – pore [g,h] | 0.627 – active material [b]<br>0.056 – binder [b]<br>0.037 – conductive carbon [b]<br>0.280 – pore [b,g] |
| Active material loading<br>$l_{am}$ / [mg/cm$^2$] | 7.776 [b]<br>(7.296 ± 0.505) | - | 12.159 [b]<br>(12.276 ± 0.130) |
| Binder loading<br>$l_{bd}$ / [mg/cm$^2$] | 0.349 [b]<br>(0.328 ± 0.040) | - | 0.436 [b]<br>(0.445 ± 0.027) |
| Conductive carbon loading<br>$l_c$ / [mg/cm$^2$] | - | - | 0.327 [b]<br>(0.328 ± 0.007) |
| Pore phase Bruggeman factor<br>$\beta_{ey}$ / [-] | 1.8 [e,[39]] ± 0.2 [f] | 2 [e,[39]] ± 0.2 [f] | 2 [e,[39]] ± 0.2 [f] |
| Electroconductive phase Bruggeman factor<br>$\beta_{el}$ / [-] | 1.5 [d,[6]] | - | 1.5 [d,[6]] |
| Symmetry factor<br>$\alpha$ / [-] | 0.5 [d,[6]] | - | 0.5 [d,[6]] |
| Kinetic constant<br>$\mathring{k}_{int}$ / [A m$^{-2}$] | 53.5750 [e,[4]] ± 0.3033 [f] | - | 9.2960 [e,[5]] ± 0.3033 [f] |
| Double layer capacitance<br>$C_{dl}$ / [F/m$^2$] | 0.803 [e] | | 0.232 [e] |
| Maximum Li concentration<br>$c_s^{max}$ / [mol m$^{-3}$] | 31360 [d,[4]] | - | 48989 [d,[40,41]] |

| | | | |
|---|---|---|---|
| Open circuit potential $E_{eq}$ / [V] | Equations (30)-(36) [e,[25,35]] | - | Equations (37)-(39) [e,[36]] |
| Characteristic interface length $a$ / [µm] | 3.1623 [d,[7]] | - | - |
| Standard potential of intercalated Li $E^{\theta}$ / [V] | 0.123 [e,[35]] | - | 3.99 [e,[36]] |
| Entropic heat coefficient $EHC$ / [V K$^{-1}$] | Figure S14 [e,[33,37]] | - | Figure S14 [d,[34,42]] |
| Min/Max state of lithiation $\tilde{c}_s^{min/max}$ / [-] | 0.021 – 0.792 [b]<br>(0.022 ± 0.001) – (0.799 ± 0.028) | - | 0.293 – 0.907 [b]<br>(0.294 ± 0.010) – (0.907 ± 0.007) |
| Effective electrical conductivity $\sigma_{ed,eff}$ / [S m$^{-1}$] | 36.12 [d,[7]] | - | 0.79 [d,[5,43]] |
| Solid-phase diffusivity $D_s^{\circ}$ / [m2 s$^{-1}$] | Figure S15 [e,[7]] | - | Figure S15 [e,[5]] |
| Charge-transfer constant activation energy $E_{k_{int,i}}$ / [J mol$^{-1}$] | 32694 [d,[6]] | - | 25000 [d,[6]] |
| Solid-phase diffusion activation energy $E_{D_{s,i}}$ / [J mol$^{-1}$] | 35000 [d,[6]] | - | 35000 [d,[6]] |
| Initial electrolyte concentration $c^{in}$ / [mol m$^{-3}$] | ← | 1000 [d,[6]] | → |
| Transference number of positive charges $t_+$ / [-] | ← | 0.27 [d,[38,44]] | → |
| Ionic conductivity $\sigma$ / [S m$^{-1}$] | ← | Equation (40) [d,[38]] | → |
| Ambipolar diffusivity $\tilde{D}$ / [m$^2$ s$^{-1}$] | ← | Equation (41) [d,[38]] | → |
| Thermodynamic factor $\gamma_{\pm}$ / [-] | ← | Equation (42) [d,[38]] | → |
| Contact resistance $R_{el,ext}$ / [mΩ m$^2$] | 1.725 charge/discharge cycles & 2.162 EIS [e] | | |
| Heat transfer coefficient $h$ / [W m$^{-2}$ K$^{-1}$] | 10 [e] | | |
| Mass of the battery with case $M$ / [g] | 42.285 [c]<br>(42.285 ± 0.047) | | |
| Equivalent specific heat $c_p$ / [J kg$^{-1}$ K$^{-1}$] | 945 [b] | | |
| Active electrode cross-sectional area $A_{act}$ / [cm$^2$] | 920.077 [c] – assumed equal for negative and positive electrodes<br>(920.077 ± 18.944) | | |
| Negative/Positive electrode cross-sectional area ratio $A_{act,N/P}$ / [-] | 1.075 [c] – updates negative electrode loadings to account for $A_{act}$ assumption<br>(1.075 ± 0.023) | | |

| | |
|---|---|
| External cooling surface<br>$A_{ext}$ / [cm$^2$] | 36.783 [c]<br>(36.783 ± 0.200) |
| Maximum capacity @ 0 V<br>$Q_M^{0V}$ / [Ah] | 2.124 [c]<br>(2.130 ± 0.009) |
| Maximum capacity @ 100% SoC<br>$Q_M$ / [Ah] | 2.064 [c]<br>(2.071± 0.013) |

(): indicates average and standard deviation values obtained from teardown and characterisation analysis

a: measured
b: calculated from teardown data
c: teardown data
d: literature data
e: initialised from the literature (with reference number) and adapted to fit to experimental data
f: determined with jackknife method applied to model calibration experimental data
g: verifies constraint on battery internal volume
h: datasheet

The following equations, tables, and plots represent the functions implemented in the model for corresponding quantities.

The open circuit potential of graphite follows the phase-field approach by Bazant and co-workers[15,16,25], adapted to match the OCP of graphite obtained from Billau et al.[35] (Figure S13):

$$E_{eq,N} = E_N^\theta - \frac{R_g T}{F}\tilde{\mu}_{td,N} \tag{30}$$

$$\tilde{\mu}_{td,N} = (A + B + C + D + E + F) \tag{31}$$

$$B = \left(-B_1 exp\left(-\frac{\tilde{c}_{s,N}}{B_2}\right) + B_3\left(tanh\left(\frac{\tilde{c}_{s,N} - B_4}{B_5}\right) - B_6\right) + \left(tanh\left(\frac{\tilde{c}_{s,N} - B_7}{B_8}\right) - B_9\right)\right)\left(-B_{10} tanh\left(\frac{\tilde{c}_{s,N} - B_{12}}{B_{13}}\right) + B_{14}\right) \tag{32}$$

$$C = -\frac{C_1}{\tilde{c}_{s,N}{}^{C_2}} \tag{33}$$

$$D = D_1\left(D_2 tanh\left(\frac{\tilde{c}_{s,N} - D_3}{D_4}\right) + D_5\right) \tag{34}$$

$$E = E_1\left(E_2 - \tilde{c}_{s,N}{}^{E_3}\right)\left(-E_4 tanh\left(\frac{\tilde{c}_s - E_5}{E_6}\right) + E_7\right)\left(E_8 tanh\left(\frac{\tilde{c}_{s,N} - E_9}{E_{10}}\right) + E_{11}\right) \tag{35}$$

$$F = \left(F_1\left(F_2 - \tilde{c}_{s,N}\right) + F_3\right)\left(F_4 tanh\left(\frac{\tilde{c}_{s,N} - F_5}{F_6}\right) + F_7\right) \tag{36}$$

**Table S5.** Graphite OCP parameters fitted to experimental data of Billaud et al.[35]

| Parameter | Value | Parameter | Value |
|---|---|---|---|
| A | 0.1800 | $D_4$ | 0.1800 |
| $B_1$ | 50.0116 | $D_5$ | 0.9999 |
| $B_2$ | 0.0161 | $E_1$ | 6.1324 |
| $B_3$ | 0.2658 | $E_2$ | 0.3793 |
| $B_4$ | 0.1460 | $E_3$ | 0.9560 |
| $B_5$ | 0.0162 | $E_4$ | 0.6360 |
| $B_6$ | 0.9775 | $E_5$ | 0.4324 |
| $B_7$ | 0.3034 | $E_6$ | 0.0153 |
| $B_8$ | 0.3776 | $E_7$ | 0.9200 |
| $B_9$ | 0.9219 | $E_8$ | 0.6360 |
| $B_{10}$ | 1.0449 | $E_9$ | 0.1967 |
| $B_{11}$ | 0.3017 | $E_{10}$ | 0.0354 |
| $B_{12}$ | 0.0940 | $E_{11}$ | 1.0782 |
| $B_{13}$ | 1.2441 | $F_1$ | 1.3265 |
| $B_{14}$ | -0.1137 | $F_2$ | 0.7222 |
| $C_1$ | 1.0767 | $F_3$ | 1.4000 |
| $C_2$ | 7.9953 | $F_4$ | 0.4200 |
| $D_1$ | 0.4193 | $F_5$ | 0.4619 |
| $D_2$ | 0.9942 | $F_6$ | 0.0200 |
| $D_3$ | 0.0354 | $F_7$ | 0.7916 |

The open circuit potential of NMC532 is taken from Zhu et al.[36] (equation (38)) and adjusted to align with the battery OCP, considering the state-of-lithiation at the electrodes derived from ICP-OES and electrical data, as well as the OCP of graphite. This adjustment involved shifting the NMC532 OCP function by a value of *sft* = 0.05 to account for irreversible Li loss (equation (37)). The adapted OCP was then compared with the NMC532 OCP obtained via GITT measurements from Verma et al.[41] (Figure

S13). Notably, this shift is consistent with the observations of Verma et al., who stated that incorporating irreversible lithium loss data results in a shift of the OCP curve, although not implemented by them. Accordingly, the reference state $\tilde{c}_s$ = 100% in Verma data corresponds to approximately 92% (ca. 0.08 $\tilde{c}_s$ loss) which is aligned with the results in this work. Then, the dimensionless chemical potential is obtained as in equation (39).

$$\tilde{c}'_{s,P} = \left(\tilde{c}_{s,P} + sft\right) \tag{37}$$

$$E_{eq,P} = p_1 * exp(p_2\, \tilde{c}'_{s,P}{}^{p_3}) + (p_4 + p_5\tilde{c}'_{s,P} + p_6\tilde{c}'_{s,P}{}^2 + p_7\tilde{c}'_{s,P}{}^3 + p_8\tilde{c}'_{s,P}{}^4 + p_9\tilde{c}'_{s,P}{}^5 + p_{10}\tilde{c}'_{s,P}{}^6 + p_{11}\tilde{c}'_{s,P}{}^7 \\ + + p_{12}\tilde{c}'_{s,P}{}^8 + p_{13}\tilde{c}'_{s,P}{}^9 + p_{14}\tilde{c}'_{s,P}{}^{10} + p_{15}\tilde{c}'_{s,P}{}^{11} + p_{16}\tilde{c}'_{s,P}{}^{12} + p_{17}\tilde{c}'_{s,P}{}^{13} + p_{18}\tilde{c}'_{s,P}{}^{14}) \tag{38}$$

$$\tilde{\mu}_{td,P} = \left(E_{eq,P} - E_P^{\theta}\right)\frac{F}{R_g T} \tag{39}$$

**Table S6.** NMC532 OCP parameters from Zhu et al.[36]

| Parameter | Value |
|---|---|
| $p_1$ | -0.0006 |
| $p_2$ | 6.5602 |
| $p_3$ | 41.4821 |
| $p_4$ | 5.3147 |
| $p_5$ | -4.1583 |
| $p_6$ | 27.2341 |
| $p_7$ | -272.4852 |
| $p_8$ | 1190.2234 |
| $p_9$ | -2073.7655 |
| $p_{10}$ | -829.7905 |
| $p_{11}$ | 8698.1128 |
| $p_{12}$ | -10743.7433 |
| $p_{13}$ | -2057.8089 |
| $p_{14}$ | 12656.3098 |
| $p_{15}$ | -1571.0943 |
| $p_{16}$ | -14557.4206 |
| $p_{17}$ | 13176.5754 |
| $p_{18}$ | -3640.1177 |

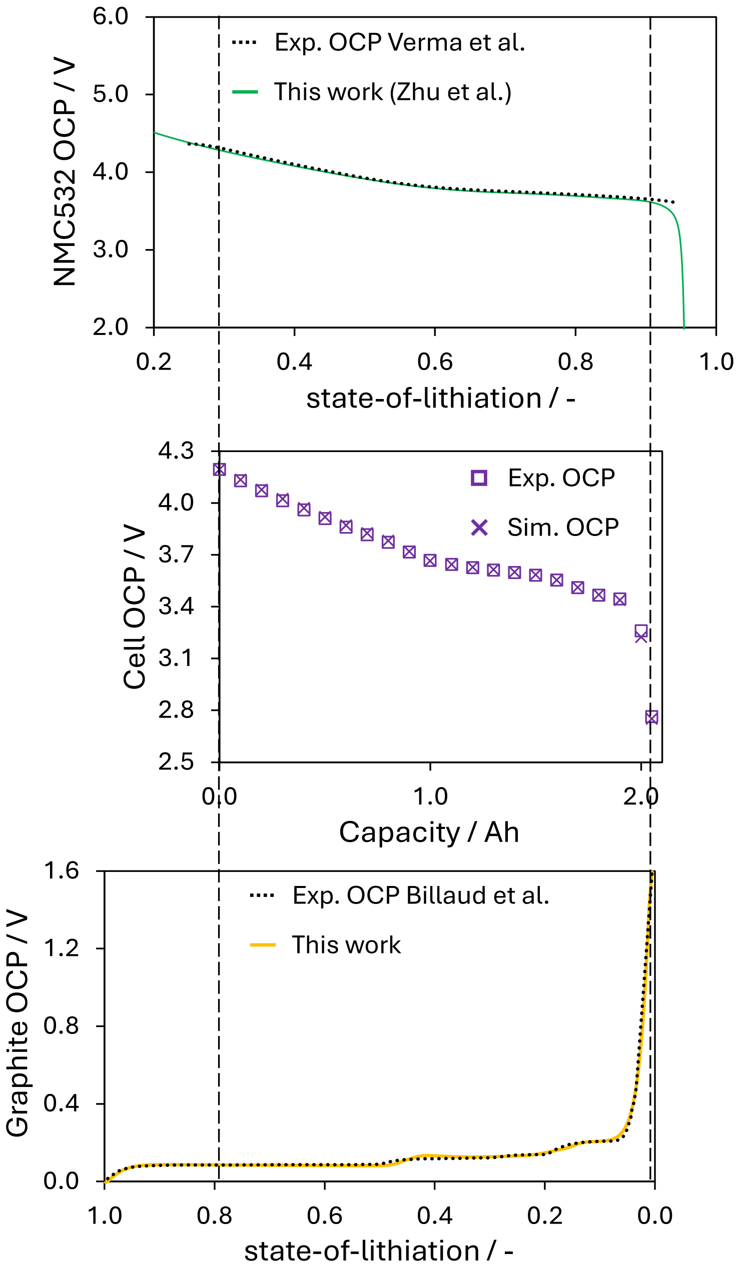


**Figure S13. Equilibrium potential (OCP) of the cell and electrodes.** The second row shows the OCP of the cell obtained via the GITTDCH protocol, where empty squares represent experimental data and x-markers indicate simulated values. Each x-marker in the second row is computed as the difference between the positive electrode OCP (first row, NMC532) and the negative electrode OCP (third row, graphite), both of which vary with the average state-of-lithiation at the electrodes. The minimum and maximum state-of-lithiation at the electrodes are identified following the approach detailed in Supplementary Figure S10. Solid lines in the first and third rows correspond to the OCP functions implemented in the model, while dotted lines represent experimental electrode OCPs[35,41].

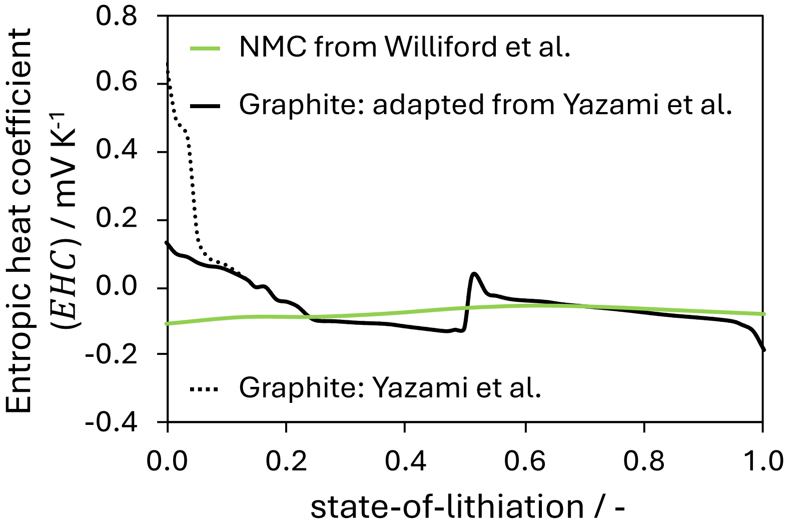


**Figure S14. Entropic heat coefficients.** For graphite, the entropic heat coefficient was fitted to constant-current data (solid line) by adapting the function of Yazami et al.[33] (dotted line), while for NMC532 the function of Williford et al.[34] was used without modification (solid and dotted lines overlap). The numerical values used in this study are listed in Tables S7 and S8 for the negative and positive electrodes, respectively.

**Table S7.** Entropic heat coefficient for graphite adapted from Yazami et al.[33]

| $\tilde{c}_{s,N}$ / - | $EHC_N$ / mV K$^{-1}$ | $\tilde{c}_{s,N}$ / - | $EHC_N$ / mV K$^{-1}$ |
|---|---|---|---|
| 0.0000 | 0.1319 | 0.4018 | -0.1172 |
| 0.0163 | 0.0989 | 0.4352 | -0.1244 |
| 0.0354 | 0.0888 | 0.4685 | -0.1308 |
| 0.0524 | 0.0718 | 0.4833 | -0.1265 |
| 0.0687 | 0.0625 | 0.4982 | -0.1237 |
| 0.0865 | 0.0589 | 0.5124 | 0.0365 |
| 0.1021 | 0.0503 | 0.5358 | -0.0179 |
| 0.1347 | 0.0222 | 0.5528 | -0.0264 |
| 0.1488 | 0.0000 | 0.5811 | -0.0372 |
| 0.1658 | 0.0000 | 0.6329 | -0.0436 |
| 0.1843 | -0.0372 | 0.6676 | -0.0522 |
| 0.2027 | -0.0443 | 0.7477 | -0.0650 |
| 0.2197 | -0.0579 | 0.8299 | -0.0815 |
| 0.2367 | -0.0851 | 0.8653 | -0.0872 |
| 0.2516 | -0.0994 | 0.9135 | -0.0943 |
| 0.2828 | -0.1015 | 0.9468 | -0.1015 |
| 0.3189 | -0.1058 | 0.9624 | -0.1115 |
| 0.3671 | -0.1094 | 0.9816 | -0.1301 |

**Table S8.** Entropic heat coefficient for NMC class of material from Williford et al.[34]

| $\tilde{c}_{s,P}$ / - | $EHC_P$ / mV K$^{-1}$ |
|---|---|
| 0.0000 | -0.1110 |
| 0.1266 | -0.0902 |
| 0.2492 | -0.0895 |
| 0.3694 | -0.0790 |
| 0.4927 | -0.0624 |
| 0.6137 | -0.0542 |
| 0.7339 | -0.0588 |
| 0.8589 | -0.0691 |
| 1.0000 | -0.0805 |

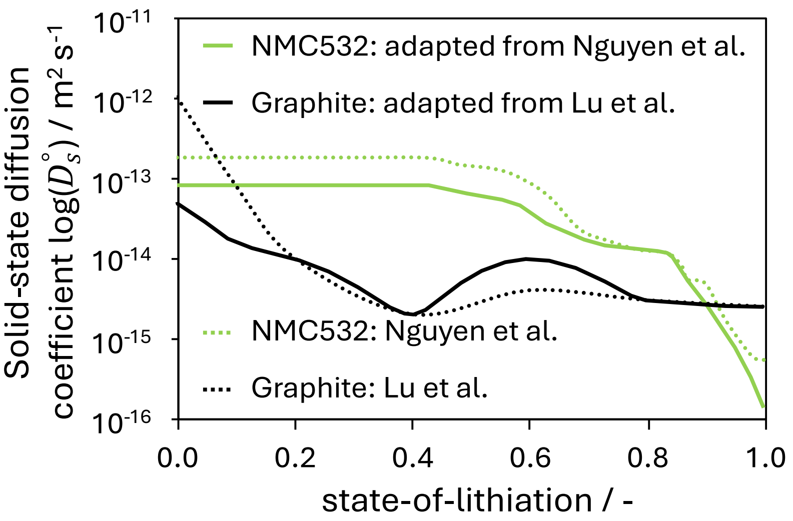


**Figure S15. Solid-state diffusion coefficients.** Diffusion coefficients obtained by fitting constant-current and EIS data using functions adapted from Lu et al.[7] and Nguyen et al.[5]. Solid lines denote the fitted functions used in this work, while dotted lines show the original literature functions. The numerical values used in this study are reported in Table S9 and Table S10 for the negative and positive electrodes, respectively.

**Table S9.** Solid-state diffusion coefficient at 25 °C of graphite as adapted from Lu et al.[7]

| $\tilde{c}_{s,N}$ / - | $\mathring{D}_{s,N}$ / $10^{-14}$ m$^2$ s$^{-1}$ |
|---|---|
| 0.00 | 5.00 |
| 0.10 | 1.60 |
| 0.20 | 1.00 |
| 0.40 | 0.20 |
| 0.60 | 1.00 |
| 0.80 | 0.30 |
| 1.00 | 0.25 |

**Table S10.** Solid-state diffusion coefficient at 25 °C of NMC532 as adapted from Nguyen et al.[5]

| $\tilde{c}_{S,P}$ / - | $D^{\circ}_{S,P}$ / $10^{-14}$ m$^2$ s$^{-1}$ |
|---|---|
| 0.000 | 8.106 |
| 0.297 | 8.106 |
| 0.426 | 8.106 |
| 0.481 | 6.630 |
| 0.530 | 5.834 |
| 0.580 | 4.685 |
| 0.631 | 2.659 |
| 0.732 | 1.443 |
| 0.785 | 1.313 |
| 0.834 | 1.176 |
| 0.859 | 0.690 |
| 0.869 | 0.530 |
| 0.931 | 0.126 |
| 0.981 | 0.029 |
| 1.000 | 0.013 |

Electrolyte transport properties used in the model come from the work of Landesfeind et al.[38,44] for 1M $LiPF_6$ in EC-EMC (3:7) with 2 % FEC which is the electrolyte assumed in this study. Equations (40)-(42) use as input variables the concentration of lithium-ions $c$ and the temperature $T$ in the units of mol m$^{-3}$ and K, respectively.

$$\sigma = 0.1 p_{1,\sigma}\left(1 + \left(T - p_{2,\sigma}\right)\right)\frac{c}{1000}\frac{\left(1 + p_{3,\sigma}\sqrt{\frac{c}{1000}} + p_{4,\sigma}\left(1 + p_{5,\sigma} exp\left(\frac{1000}{T}\right)\frac{c}{1000}\right)\right)}{1 + \left(\frac{c}{1000}\right)^4\left(p_{6,\sigma} exp\left(\frac{1000}{T}\right)\right)} \tag{40}$$

$$\tilde{D} = p_{1,D} exp\left(p_{2,D}\frac{c}{1000}\right) exp\left(\frac{p_{3,D}}{T}\right) exp\left(\frac{p_{4,D}}{T}\frac{c}{1000}\right) 10^{-10} \tag{41}$$

$$\gamma_{\pm} = \left(p_{1,\gamma} + p_{2,\gamma}\frac{c}{1000} + p_{3,\gamma}T + p_{4,\gamma}\left(\frac{c}{1000}\right)^2 + p_{5,\gamma}\frac{c}{1000}T + p_{6,\gamma}T^2 + p_{7,\gamma}\left(\frac{c}{1000}\right)^3 + p_{8,\gamma}T\left(\frac{c}{1000}\right)^2 + p_{9,\gamma}T^2\frac{c}{1000}\right) \tag{42}$$

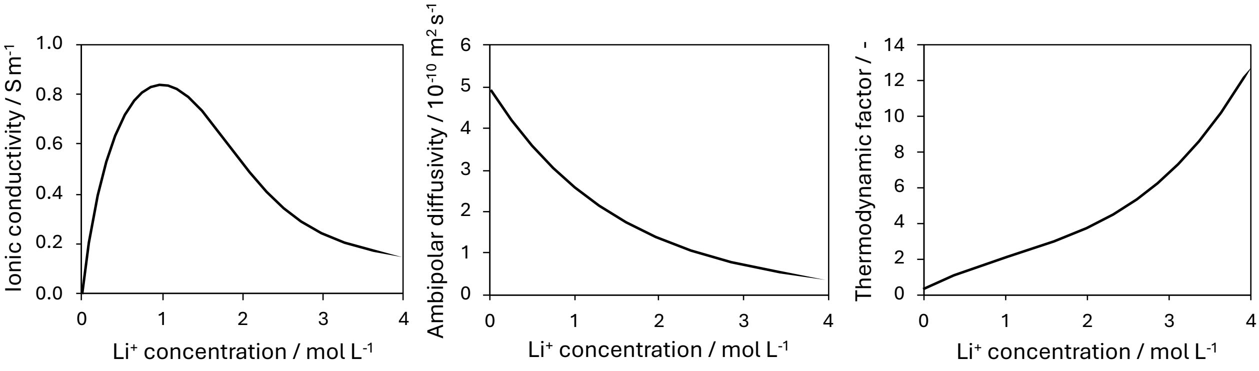


**Figure S16. Transport properties of the electrolyte.** The electrolyte solution properties from the work of Landesfeid et al.[31,38] evaluated at 20 °C. The functions and their parameters are reported in Equations (40)-(42) and Table S11.

**Table S11.** Parameters required for electrolyte transport properties from Landesfeind et al. [31,38]

| Ionic conductivity $\sigma$ / S m$^{-1}$ | | Ambipolar diffusivity $\widetilde{D}$ / m$^2$ s$^{-1}$ | | Thermodynamic factor $\gamma_{\pm}$ / - | |
|---|---|---|---|---|---|
| Parameter | Value | Parameter | Value | Parameter | Value |
| $p_{1,\sigma}$ | 0.52100 | $p_{1,D}$ | 1010.0000 | $p_{1,\gamma}$ | 25.700000 |
| $p_{2,\sigma}$ | 228.00000 | $p_{2,D}$ | 1.0100 | $p_{2,\gamma}$ | -45.100000 |
| $p_{3,\sigma}$ | -1.06000 | $p_{3,D}$ | -1560.0000 | $p_{3,\gamma}$ | -0.177000 |
| $p_{4,\sigma}$ | 0.35300 | $p_{4,D}$ | -487.0000 | $p_{4,\gamma}$ | 1.940000 |
| $p_{5,\sigma}$ | -0.00359 | | | $p_{5,\gamma}$ | 0.295000 |
| $p_{6,\sigma}$ | 0.00148 | | | $p_{6,\gamma}$ | 0.000308 |
| | | | | $p_{7,\gamma}$ | 0.259000 |
| | | | | $p_{8,\gamma}$ | -0.009460 |
| | | | | $p_{9,\gamma}$ | -0.000454 |

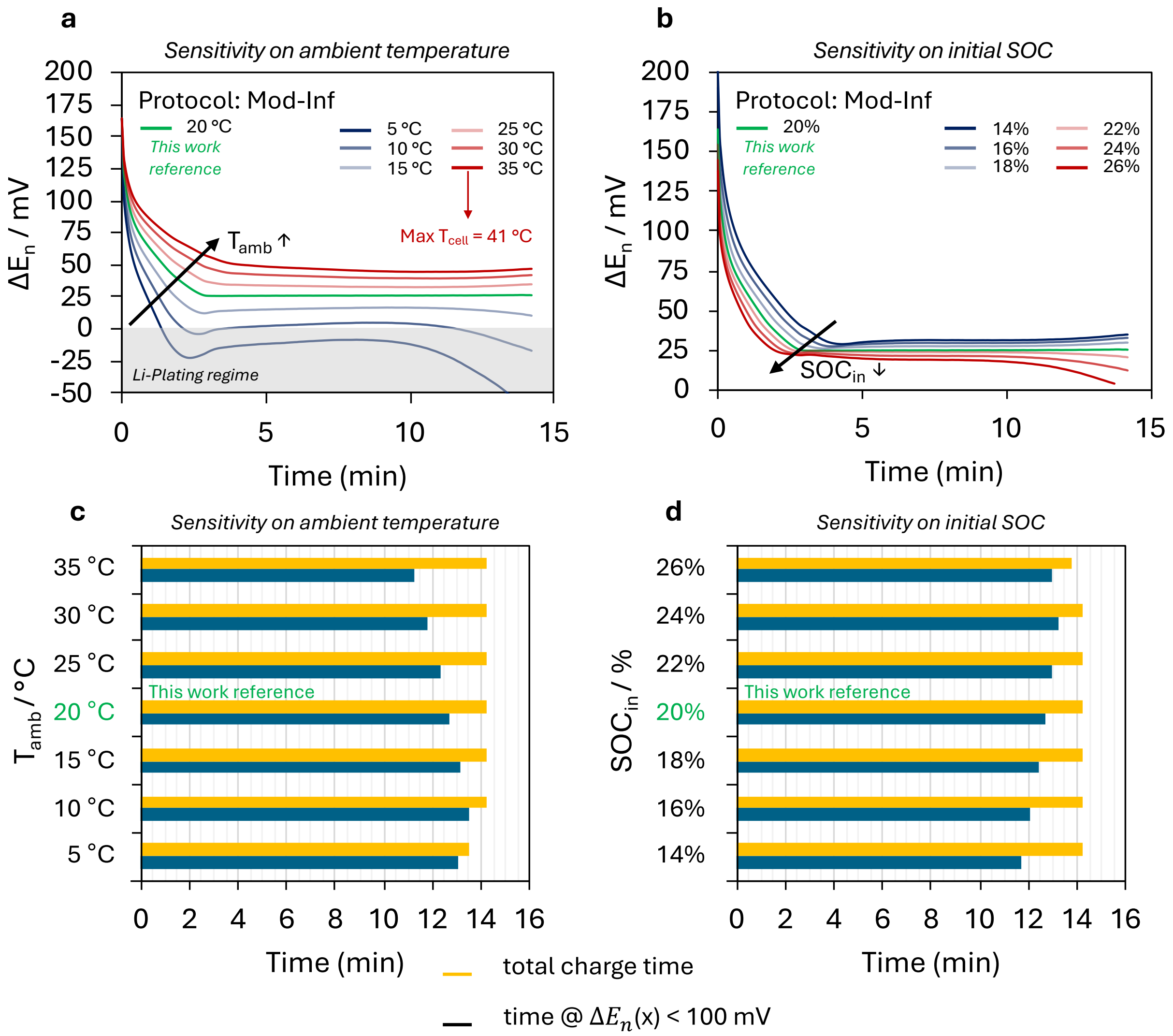


**Figure S17. Sensitivity analysis to initial state-of-charge and ambient temperature.** a,b) Evolution of the minimum potential difference at the negative electrode–separator interface ($\Delta E_n$) during charging with the Mod-Inf protocol for different ambient temperatures (a, 5–35 °C) and initial SOC values (b, 14–26%). Reference conditions (20 °C, 20% SOC) are highlighted in green. Note that the highest temperature reached by the cell is 41 °C when $T_{amb}$ = 35 °C is simulated. c,d) Corresponding total charging time (yellow) and time spent with $\Delta E_n$ < 100 mV (blue) for the same cases as in a,b).

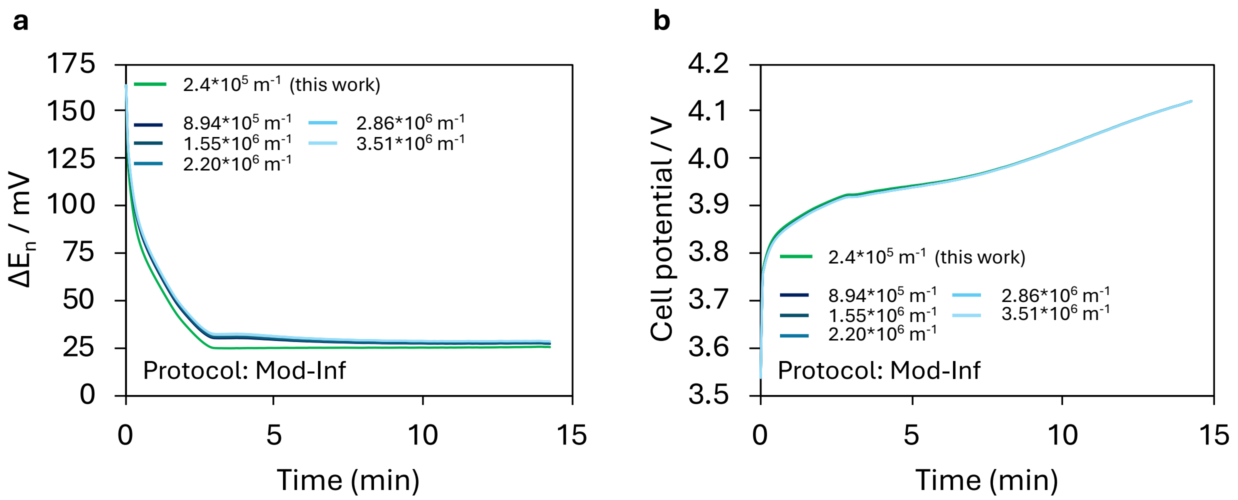


**Figure S18. Sensitivity analysis to graphite specific surface per unit of electrode volume.** Prediction of the minimum potential difference at the negative electrode–separator interface, $\Delta E_n$ (a), and the cell potential (b) during charging with the Mod-Inf protocol for different values of specific surface area ($A_{am}$). The reference value used in this work (2.4 × $10^5$ $m^{-1}$, green) is compared with values up to ca. one order of magnitude higher, consistent with other literature studies[4,25].

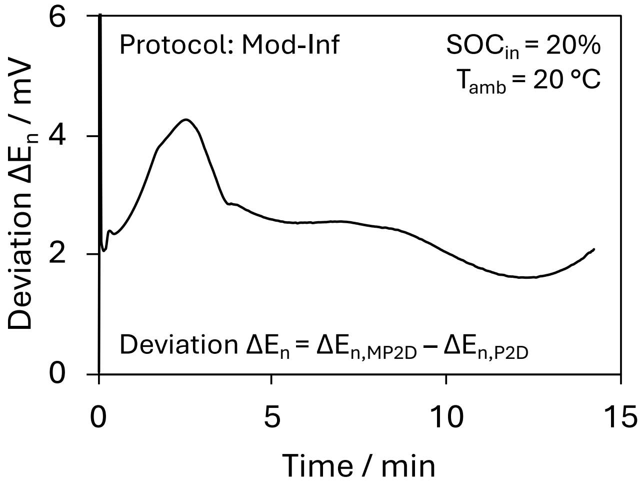


**Figure S19. Deviation of $\Delta E_n$ between P2D and multi-particle P2D (MP2D).** Simulated $\Delta E_n$ deviation during charging under the Mod-Inf protocol, comparing the standard P2D model with an MP2D implementation incorporating a particle size distribution (PSD). The PSD, derived from Supplementary Figure S11, is discretised into three bins with particle radii of 5.08 µm, 8.19 µm and 12.06 µm and corresponding volume fractions of 0.113, 0.743 and 0.144, respectively; the discretisation preserves the same volume-weighted effective radius as used in the single-radius P2D model.

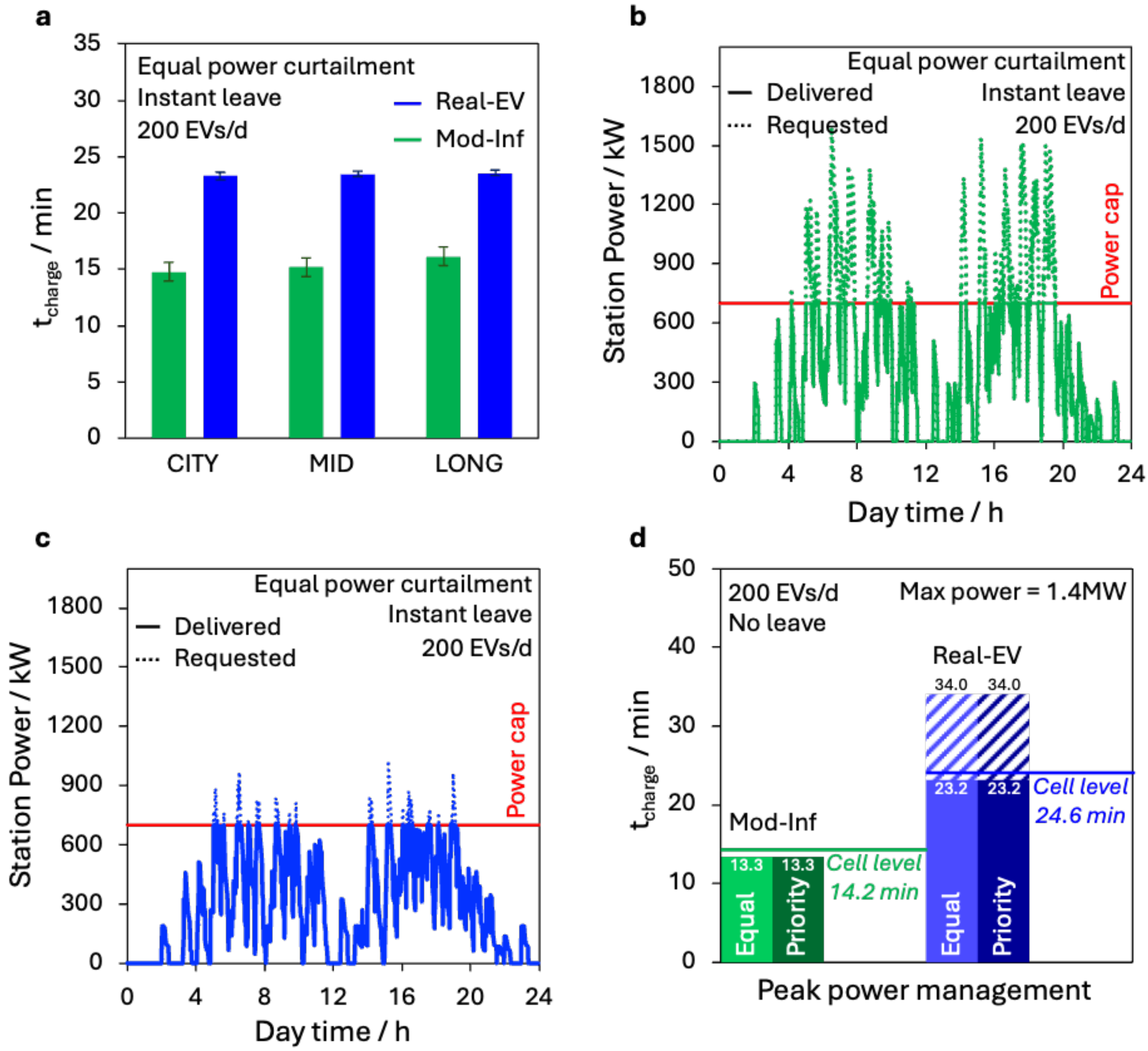


**Figure S20. System level comparison of Mod-Inf and Real-EV fast-charge protocols via Monte Carlo analysis.** (a) Total charging times for each EV class. (b, c) Requested (dotted) and delivered (solid) station power profiles under Mod-Inf (b) and Real-EV (c) protocols; the station power cap (700 kW) is shown in red. (d) Comparison of total charging time at 200 EVs/day, assuming no leave if sockets are occupied, of the two power management strategies: equal power curtailment (Equal, light colours) and priority-based charge by arrival time (Priority, dark colours). The station maximum power is raised to 1.4 MW. Solid bars report the actual charge time, while striped bars indicate queue time for Mod-Inf and Real-EV fast-charge protocols. Dashed lines represent cell-level charging times.

## Data availability

All battery datasets containing raw experimental data are available at the following repository (10.17632/jnnxmt35sy.1). Source data are provided with the paper.

## References


[1] F. Ciucci, *Curr Opin Electrochem* **2019**, *13*, 132.

[2] James E. Gentle, *Random Number Generation and Monte Carlo Methods*, 2nd ed., Springer New York, NY, New York **2003**.

[3] T. Li, X.-Z. Yuan, L. Zhang, D. Song, K. Shi, C. Bock, *Electrochemical Energy Reviews* **2020**, *3*, 43.

[4] S. Ahn, M. Lagnoni, Y. Yuan, A. Ogarev, E. Vavrinyuk, G. Voynov, E. Barrett, A. Pelli, A. Atrashchenko, A. Platonov, S. Gurevich, M. Gorokhov, D. Rupasov, A. W. Robertson, R. A. House, L. R. Johnson, A. Bertei, D. V. Chernyshov, *ACS Appl Energy Mater* **2023**, *6*, 8455.

[5] T.-T. Nguyen, B. Delobel, M. Berthe, B. Fleutot, A. Demortière, C. Delacourt, *J Electrochem Soc* **2022**, *169*, 040546.

[6] M. Lagnoni, C. Nicolella, A. Bertei, *Electrochim Acta* **2021**, *394*.

[7] X. Lu, M. Lagnoni, A. Bertei, S. Das, R. E. Owen, Q. Li, K. O'Regan, A. Wade, D. P. Finegan, E. Kendrick, M. Z. Bazant, D. J. L. Brett, P. R. Shearing, *Nat Commun* **2023**, *14*, 5127.

[8] M. Lagnoni, C. Scarpelli, G. Lutzemberger, A. Bertei, *J Energy Storage* **2024**, *94*, 112326.

[9] M. Lagnoni, G. Armiento, C. Nicolella, A. Bertei, *Progress in Energy* **2024**, *6*, 023002.

[10] J. Newman, W. Tiedemann, *AIChE Journal* **1975**, *21*, 25.

[11] F. Ciucci, W. Lai, *Transp Porous Media* **2011**, *88*, 249.

[12] M. Lagnoni, C. Nicolella, A. Bertei, *J Electrochem Soc* **2022**, *169*, 020570.

[13] J. Newman, K. E. Thomas, H. Hafezi, D. R. Wheeler, *J Power Sources* **2003**, *119–121*, 838.

[14] A. J. Bard, L. R. Faulkner, *Electrochemical Methods: Fundamentals and Applications*, 2nd ed., Wiley, New York **2001**.

[15] M. Z. Bazant, *Acc Chem Res* **2013**, *46*, 1144.

[16] R. B. Smith, M. Z. Bazant, *J Electrochem Soc* **2017**, *164*, E3291.

[17] T. R. Ferguson, M. Z. Bazant, *J Electrochem Soc* **2012**, *159*, A1967.

[18] R. B. Smith, E. Khoo, M. Z. Bazant, *The Journal of Physical Chemistry C* **2017**, *121*, 12505.

[19] J. S. Horner, G. Whang, D. S. Ashby, I. V. Kolesnichenko, T. N. Lambert, B. S. Dunn, A. A. Talin, S. A. Roberts, *ACS Appl Energy Mater* **2021**, *4*, 11460.

[20] A. Bertei, A. Lamorgese, R. Mauri, *Journal of Non-Equilibrium Thermodynamics* **2020**, *45*, 27.

[21] J. W. Cahn, J. E. Hilliard, *J Chem Phys* **1958**, *28*, 258.

[22] B. C. Han, A. Van der Ven, D. Morgan, G. Ceder, *Electrochim Acta* **2004**, *49*, 4691.

[23] S. Das, P. M. Attia, W. C. Chueh, M. Z. Bazant, *J Electrochem Soc* **2019**, *166*, E107.

[24] B. Horstmann, F. Single, A. Latz, *Curr Opin Electrochem* **2019**, *13*, 61.

[25] H. Lian, M. Z. Bazant, *J Electrochem Soc* **2024**, *171*, 010526.

[26] X.-G. Yang, Y. Leng, G. Zhang, S. Ge, C.-Y. Wang, *J Power Sources* **2017**, *360*, 28.

[27] M. Barbieri, M. Ceraolo, G. Lutzemberger, C. Scarpelli, T. Pesso, M. Giovannucci, *2020 AEIT International Conference of Electrical and Electronic Technologies for Automotive (AEIT AUTOMOTIVE)* **2020**, 1.

[28] R. Franke-Lang, J. Kowal, *Modelling* **2021**, *2*, 259.

[29] A. Frank, J. Sturm, M. Steinhardt, A. Rheinfeld, A. Jossen, *ECS Advances* **2022**, *1*, 40502.

[30] D. Bernardi, E. Pawlikowski, J. Newman, *J Electrochem Soc* **1985**, *132*, 5.

[31] A. Ehrl, J. Landesfeind, W. A. Wall, H. A. Gasteiger, *J Electrochem Soc* **2017**, *164*, A826.

[32] M. Ebner, D.-W. Chung, R. E. García, V. Wood, *Adv Energy Mater* **2014**, *4*, 1301278.

[33] R. Yazami, Y. Reynier, *J Power Sources* **2006**, *153*, 312.

[34] R. E. Williford, V. V. Viswanathan, J. G. Zhang, *J Power Sources* **2009**, *189*, 101.

[35] D. Billaud, F. X. Henry, *Solid State Commun* **2002**, *124*, 299.

[36] H. Zhu, T. A. P. Evans, P. J. Weddle, A. M. Colclasure, B.-R. Chen, T. R. Tanim, T. L. Vincent, R. J. Kee, *J Electrochem Soc* **2024**, *171*, 050512.

[37] J. Sturm, A. Rheinfeld, I. Zilberman, F. B. Spingler, S. Kosch, F. Frie, A. Jossen, *J Power Sources* **2019**, *412*, 204.

[38] J. Landesfeind, H. A. Gasteiger, *J Electrochem Soc* **2019**, *166*, A3079.

[39] A. M. Colclasure, A. R. Dunlop, S. E. Trask, B. J. Polzin, A. N. Jansen, K. Smith, *J Electrochem Soc* **2019**, *166*, A1412.

[40] S. Yang, X. Wang, X. Yang, Y. Bai, Z. Liu, H. Shu, Q. Wei, *Electrochim Acta* **2012**, *66*, 88.

[41] A. Verma, K. Smith, S. Santhanagopalan, D. Abraham, K. P. Yao, P. P. Mukherjee, *J Electrochem Soc* **2017**, *164*, A3380.

[42] F. Yun, W. Jin, L. Tang, W. Li, J. Pang, S. Lu, *J Electrochem Soc* **2016**, *163*, A639.

[43] R. Amin, Y.-M. Chiang, *J Electrochem Soc* **2016**, *163*, A1512.

[44] A. Ehrl, J. Landesfeind, W. A. Wall, H. A. Gasteiger, *J Electrochem Soc* **2017**, *164*, A2716.

## Nomenclature

*Abbreviations*

CC – Current collector

EIS – Electrochemical impedance spectroscopy

EV – Electric vehicle

GITT – Galvanostatic intermittent titration technique

ICP-OES – Inductively coupled plasma optical emission spectroscopy

Man-Max – Manufacturer maximum charge protocol

Mod-Inf – Model-informed fast-charge protocol

MP2D – Multi-particle pseudo-2-dimensional model

NMC532 – Nickel manganese cobalt oxide ($LiNi_{0.5}Mn_{0.3}Co_{0.2}O_2$)

OCP – Open circuit potential

PSD – Particle size distribution

Real-EV – Commercial fast-charge protocol

SEI – Solid electrolyte interphase

SEM – Scanning electron microscopy

SoC – State-of-charge

STDCHA – Standard charge

REFDCH – Reference discharge

TGA – Thermogravimetric analysis

RMSE – Root mean square error

*Latin Symbols*

$A$ – Surface area (m$^2$)

$A_{act}$ – Active cross-sectional area (m$^2$)

$A_{am}$ – Specific active material surface area per electrode volume (m$^{-1}$)

$A_{ext}$ – External cooling surface area (m$^2$)

$A_{geo}$ – Measured cross-sectional area (m$^2$)

$c$ – Concentration (mol m$^{-3}$)

$c^{in}$ – Initial electrolyte concentration (mol m$^{-3}$)

$c_p$ – Specific heat of the battery (J kg$^{-1}$ K$^{-1}$)

$c_s$ – Concentration of intercalated lithium in active material (mol m$^{-3}$)

$\tilde{c}_s$ – State-of-lithiation in active material (-)

$c_s^{max}$ – Maximum lithium concentration in active material (mol m$^{-3}$)

$C_{dl}$ – Double-layer capacitance (F m$^{-2}$)

$D_s$ – Solid-state diffusion coefficient (m$^2$ s$^{-1}$)

$e_{tf}$ – Impedance relative error at given frequency (-)

$EHC$ – Entropic heat coefficient (V K$^{-1}$)

$E_{D_s}$ – Activation energy for solid-state diffusion coefficient (J mol$^{-1}$)

$E_{eq}$ – Equilibrium potential (V)

$E_{k_{int}}$ – Activation energy for charge-transfer reaction (J mol$^{-1}$)

$E^{\theta}$ – Standard equilibrium potential (V)

$f$ – frequency (Hz)

$F$ – Faraday constant (C mol$^{-1}$)

$i_{app}$ – Applied current (A)

$i_{pert}$ – EIS perturbation current (A)

$J_{int}$ – Intercalation current density (A m$^{-2}$)

$J_{conc}$ – Diffusional flux of $Li^+$ in electrolyte (mol m$^{-2}$ s$^{-1}$)

$J_1$ – Electronic current density in electrode (A m$^{-2}$)

$J_2$ – Ionic current density in electrolyte (A m$^{-2}$)

$l$ – Mass loading (mg cm$^{-2}$)

$L$ – Electrode thickness (μm)

$L_{tot}$ – Total thickness of battery cell (μm)

$M_{cell} \equiv M$ – Mass of the battery (kg)

$n$ – Moles of a given species (mol)

$N_s$ – Li molar flux in active material (mol m$^{-2}$ s$^{-1}$)

$\dot{q}_{gen}$ – Total volumetric heat generation (W m$^{-3}$)

$\dot{q}_{rev}$ – Reversible heat generation (W m$^{-3}$)

$\dot{q}_{rxn}$ – Irreversible reaction heat (W m$^{-3}$)

$\dot{q}_{ohm}$ – Ohmic heat generation (W m$^{-3}$)

$Q_M$ – Capacity at 100% SoC (Ah)

$Q_M^{0V}$ – Capacity at 0 V (Ah)

$r_p$ – Particle radius (μm)

$R_{el,ext}$ – External electrical resistance (Ω m$^2$)

$R_g$ – Universal gas constant (J mol$^{-1}$ K$^{-1}$)

$T$ – Temperature (K)

$T_{amb}$ – Ambient temperature (K)

$t_+$ – $Li^+$ transference number (-)

$V$ – Battery voltage (V)

$w$ – Weight fraction (-)

$y$ – Radial coordinate in active material particle (m)

$x$ – Through-thickness coordinate (m)

$Z_{exp}$ – Experimental impedance (Ω)

$Z_{sim}$ – Simulated impedance (Ω)

*Greek Symbols*

$\alpha$ – Symmetry factor (-)

$\beta$ – Bruggeman coefficient (-)

$\gamma_{\pm}$ – Thermodynamic factor (-)

$\Delta E$ – Electrochemical potential difference (V)

$\eta_{int}$ – Activation overpotential (V)

$\varepsilon$ – Volume fraction (-)

$\varepsilon_{ey}$ – Porosity or electrolyte volume fraction (-)

$\mu$ – Chemical potential (J mol$^{-1}$)

$\tilde{\mu}$ – Dimensionless chemical potential (-)

$\tilde{\mu}^{*}$ – Reduced electrochemical potential (V)

$\rho$ – Density (kg m$^{-3}$)

$\sigma$ – Conductivity (S m$^{-1}$)

$\tau$ – Tortuosity factor (-)

*Subscripts*

*amb* – Ambient

*act* – Active (electrode or material)

*am* – Active material

*bd* – Binder

*c* – Conductive carbon

*dl* – Double-layer

*ed (el)* – Electron conducting domain

*eff* – Effective property

*ey* – Electrolyte phase

*exp* – Experimental

*ext* – External

*gen* – Generated (heat)

*gr* – Graphite

*in* – Referred to the internal separator

*int* – Intercalation

*max/min* – Maximum/Minimum

*N* – Negative electrode

*out* – Referred to the outer separator

*P* – Positive electrode

*rev* – Reversible

*rxn* – Reaction

*s* – Solid active material

*S* – Separator

*sim* - Simulated

*tot* – Total

*Superscripts*

° – Reference condition

*0V* – At zero voltage state

* – Reduced electrochemical potential

*eff* – Effective property

*ext* – External property

*max/min* – Maximum/Minimum state

$\theta$ – Standard state
